# A Fusion Multi-Strategy Gray Wolf Optimizer for Enhanced Coverage Optimization in Wireless Sensor Networks

**DOI:** 10.3390/s25175405

**Published:** 2025-09-02

**Authors:** Zhenkun Liu, Yun Ou, Zhuo Yang, Shuanghu Wang

**Affiliations:** 1 School of Communication and Electronic Engineering, Jishou University, Jishou 416000, China; 2023403897@stu.jsu.edu.cn (Z.L.); 2022405739@stu.jsu.edu.cn (S.W.); 2 School of Transportation, Changsha University of Science and Technology, Changsha 410114, China; yangzhuo@stu.csust.edu.cn

**Keywords:** wireless sensor networks, coverage optimization, gray wolf optimizer, swarm intelligence, Internet of Things, sensors

## Abstract

Wireless sensor networks (WSNs) are fundamental to applications in the Internet of Things, smart cities, and environmental monitoring, where coverage optimization is critical for maximizing monitoring efficacy under constrained resources. Conventional approaches often suffer from low global coverage efficiency, high computational overhead, and a tendency to converge to local optima. To address these challenges, this study proposes the fusion multi-strategy gray wolf optimizer (FMGWO), an advanced variant of the Gray Wolf Optimizer (GWO). FMGWO integrates various strategies: electrostatic field initialization for uniform population distribution, dynamic parameter adjustment with nonlinear convergence and differential evolution scaling, an elder council mechanism to preserve historical elite solutions, alpha wolf tenure inspection and rotation to maintain population vitality, and a hybrid mutation strategy combining differential evolution and Cauchy perturbations to enhance diversity and global search capability. Ablation studies validate the efficacy of each strategy, while simulation experiments demonstrate FMGWO’s superior performance in WSN coverage optimization. Compared to established algorithms such as PSO, GWO, CSA, DE, GA, FA, OGWO, DGWO1, and DGWO2, FMGWO achieves higher coverage rates with fewer nodes—up to 98.63% with 30 nodes—alongside improved convergence speed and stability. These results underscore FMGWO’s potential as an effective solution for efficient WSN deployment, offering significant implications for resource-constrained optimization in IoT and edge computing systems.

## 1. Introduction

In recent years, significant advancements in the fields of artificial intelligence (AI) and microelectronics have contributed to the pervasive adoption of wireless sensor networks (WSNs) in various applications, including the Internet of Things (IoT), smart cities, and environmental monitoring [1]. WSNs facilitate the operation of intelligent systems by collecting real-time data, including temperature, humidity, and chemical concentration, from distributed sensor nodes [2]. Moreover, in dynamic scenarios such as disaster response, military reconnaissance, or adaptive environmental monitoring, WSNs must rapidly adjust to changing conditions, necessitating mobile sensor nodes capable of dynamic deployment. However, the coverage optimization problem persists as a pivotal challenge in WSN design, namely, how to maximize the coverage of the monitoring area while ensuring connectivity and energy efficiency under the constraints of node number, energy, and computational resources. This problem directly impacts the accuracy of monitoring and the efficiency of resources in WSNs, especially in dynamic and heterogeneous environments, thereby limiting their reliability in intelligent networks [3]. The optimization of WSN coverage is imperative to enhance network performance and facilitate the extensive implementation of IoT applications [4].

Conventional optimization methodologies often prove challenging when it comes to addressing the intricacies inherent in the WSN coverage problem. Static deployment strategies, while effective in controlled environments, struggle to adapt to environmental changes or node failures, limiting their flexibility in real-world applications. In contrast, dynamic deployment enables sensor nodes to reposition themselves, enhancing network flexibility and robustness under resource constraints. Swarm intelligence algorithms offer a novel approach to addressing this challenge. The efficacy of Particle Swarm Optimization (PSO) in optimizing node positions is contingent upon swarm collaboration, yet its performance is constrained by the speed of convergence [5]. Ant Colony Optimization (ACO) is predicated on the principle of colony collaboration to optimize node locations; however, it is constrained by high computational overhead [6]. Deep reinforcement learning has demonstrated remarkable proficiency in dynamic optimization; however, its substantial resource requirements impose significant limitations on its practical applications [7]. The deficiencies of these methodologies underscore the pressing necessity for efficient and robust algorithms to address the high-dimensional search space and real-time constraints in WSN coverage problems.

In order to address these practical issues, a thorough analysis of the particular requirements for optimizing the coverage of WSNs was conducted. This analysis revealed that the gray wolf optimization (GWO) algorithm offers an effective optimization framework by emulating the collaborative behavior of wolves. However, the GWO algorithm is subject to certain limitations, including uneven limitations and an imbalance between exploration and exploitation, as well as local optimal traps, arising from random initialization and exploration–exploitation imbalance [8]. Therefore, in response to the pressing need to address the challenge of optimizing network coverage, we propose a fusion multi-strategy gray wolf optimizer (FMGWO), which significantly improves search efficiency and stability through electrostatic field initialization, dynamic parameter tuning, an elder council mechanism, head wolf tenure checking and a rotation mechanism, and a hybrid mutation strategy. This approach directly addresses the inefficiency and instability of existing techniques in complex WSN scenarios and achieves higher coverage with fewer nodes, outperforming algorithms such as PSO, GWO, CSA, DE, GA, FA, OGWO, DGWO1, and DGWO2. FMGWO’s innovation not only improves the performance of WSN deployments but also provides a reference framework for the IoT, edge computing, and efficient smart systems, providing a reference framework for optimization and demonstrating its potential for application in optimization problems.

## 2. Related Work

The issue of coverage in WSNs represents a fundamental challenge in various application areas, including the IoT, smart cities, and environmental monitoring. In these contexts, the objective is to optimize the coverage area while ensuring network connectivity and energy efficiency within the limitations imposed by the number of nodes, energy resources, and computational capabilities [9]. A plethora of studies have been conducted on this issue, proposing a variety of optimization methods, primarily focusing on two strategies: deterministic and random deployment [10].

Deterministic deployment is a methodical process that ensures comprehensive area coverage and network connectivity by meticulously positioning sensor nodes at predefined locations. This approach is particularly well suited for scenarios where environmental information is known, such as industrial monitoring or smart buildings [11]. Boualem A et al. have proposed a minimum semi-deterministic deployment model for mobile wireless sensor networks based on Pick’s theorem. This model effectively reduces node mobility and enhances network lifetime and coverage efficiency [12]. Zrelli A et al. investigated the k-coverage and connectivity of WSNs in border monitoring. They explored the optimal model under deterministic deployment to determine the minimum number of sensors. They also proposed a hybrid WSN scheme for Tunisian–Libyan border monitoring to achieve efficient connectivity and event detection [13]. However, deterministic deployment is contingent on detailed environmental information obtained in advance and is challenging to implement in dynamic or unknown environments, thereby limiting its flexibility and practical application [14].

Conversely, random deployment does not necessitate any a priori environmental data, and the deployment process is straightforward and adaptable, making it well suited to large-scale or emergency scenarios, such as forest fire monitoring or battlefield reconnaissance [15]. The random scattering of nodes, a deployment strategy that aims to reduce complexity, often results in coverage blindness or excessive node clustering due to an uneven distribution of nodes. This, in turn, affects coverage and connectivity [16]. In order to address these issues, researchers have devised post-optimization techniques to enhance the coverage effect by adjusting node positions. Furthermore, they have conducted theoretical analyses of the optimal and worst coverage scenarios and have proposed various improvement algorithms. These algorithms have the potential to alleviate the coverage unevenness problem to a certain extent [17]. However, random deployment continues to encounter challenges, including node redundancy and energy expenditure. Consequently, the development of more efficient deployment optimization schemes is imperative [18].

Beyond static placement problems, recent research has increasingly addressed dynamic coverage scenarios where sensors are mobile or environmental conditions change over time [19]. Approaches to dynamic coverage often rely on adaptive re-deployment, online control policies, or evolutionary algorithms that can handle time-varying objectives [20]. In particular, multi-objective evolutionary algorithms and multi-task learning frameworks have been proposed to handle conflicting goals and exploit knowledge transfers between related tasks or time instances [21]. Such methods can include interval-based uncertainty handling, inverse-mapping techniques for rapid re-initialization, or learning-based surrogates to accelerate solution updates [22]. While these dynamic and multi-objective methods are powerful for mobile or uncertain WSNs, they typically involve more complex objective formulations, additional communication for sensor mobility, or a higher computational burden [23].

In light of the limitations of conventional optimization methodologies, swarm intelligence algorithms offer a novel approach to WSN coverage optimization due to their capacity to generate complex intelligent behaviors through local interactions of simple individuals. Song J et al. proposed an enhanced algorithm, NEHPO, based on the prey–predator optimization (HPO) algorithm to address the challenges posed by the low search accuracy of HPO and its propensity to converge on a local optimum, with the objective of enhancing WSN coverage performance [24]. Although NEHPO improves the overall search capability, its hybrid structure introduces additional parameter-tuning complexity and increases computational overhead during each iteration. Furthermore, in large-scale WSN deployments, the algorithm may still suffer from unstable convergence behavior due to sensitivity to initial population distribution, and the improvements in local exploitation do not fully eliminate the tendency to stagnate when facing multimodal search landscapes.

Wang J et al. proposed an algorithm for optimizing network coverage based on the Improved Salpa Swarm Intelligence Algorithm (ATSSA). This algorithm enhances the global and local searching ability through the use of a tent chaotic sequence initialization of the population, a T-distribution variation, and an adaptive position-updating formula. The purpose of these elements is to improve WSN coverage and reduce node mobile energy consumption [25]. Despite these enhancements, ATSSA’s reliance on chaotic sequence initialization may result in excessive randomness when the search space is very large, potentially causing inefficient early-stage exploration. The incorporation of a T-distribution variation, while beneficial for avoiding premature convergence, can produce large and unpredictable position jumps that slow convergence when the search approaches near-optimal regions. Additionally, the adaptive update mechanism adds computational complexity and requires careful parameter calibration to maintain a suitable balance between exploration and exploitation across different network scales.

Chen X et al. proposed a hybrid butterfly–predator optimization (HBPO) algorithm with a dynamic quadratic parameter adaptive strategy, and the hybrid butterfly–beluga optimization algorithm (NHBBWO), which combines the advantages of beluga and butterfly optimization algorithms to improve WSN node coverage area and reduce redundancy [26]. Although HBPO and NHBBWO benefit from hybridization by integrating complementary search mechanisms, this combination inherently increases algorithmic complexity, leading to longer computation times per iteration. The performance gain is also highly dependent on appropriate parameter weighting between the two embedded metaheuristics, which reduces robustness in dynamically changing WSN environments. Moreover, hybrid algorithms may suffer from redundant search behavior when the optimization process lacks effective cooperation control, thus causing wasted computational effort and reduced scalability in large-scale deployments.

Despite the advances made by metaheuristic algorithms, such as the Hungarian method and Salpa, persistent challenges remain across most swarm intelligence-based coverage optimization methods. The majority encounter performance degradation in high-dimensional or large-scale networks, with slow convergence rates and an increased computational overhead from complex parameter tuning. Their search behaviors tend to collapse prematurely toward local optima, especially in rugged or multimodal landscapes. In mobile node scenarios, frequent relocation amplifies energy consumption, limiting the real-world applicability of these solutions. Notably, population diversity often decreases rapidly in the early search stages, reducing the algorithm’s global search capacity. These limitations impede the efficacy of node optimization deployment. These limitations have prompted researchers to investigate more efficient swarm intelligence algorithms to address the complexity of WSN coverage optimization.

To address these limitations, the GWO, introduced by Mirjalili et al., has emerged as a competitive alternative [27]. Inspired by the social hierarchy and hunting behavior of grey wolf packs, GWO provides an efficient metaheuristic optimization framework by simulating the wolf pack collaboration mechanism [27]. In comparison with algorithms such as HPO and Salpa, GWO demonstrates superiority in the optimization of WSN coverage with its simplicity and reduced computational cost, thereby effectively enhancing the coverage rate [28]. However, the performance of GWO is limited due to the uneven distribution of the initial population, the lack of flexibility in the linear convergence strategy, and the tendency to fall into local optimization in complex, high-dimensional search spaces [29].

Specifically, populations that are randomly initialized may result in inadequate coverage of the solution space [30], linear convergence factors encounter challenges in achieving a balance between global exploration and local exploitation [31], and a single-head wolf guidance mechanism may lead to convergence stagnation in multi-peak optimization scenarios [32]. These issues are more prominent in the WSN optimization coverage problem, which affects the robustness of the algorithm and the actual deployment effect to a certain extent.

In summary, deterministic and random deployment strategies offer their own advantages in terms of WSN coverage optimization. However, the limitations of these strategies highlight the necessity of advanced optimization techniques. As a class of advanced optimization techniques, swarm intelligence algorithms provide effective solutions for coverage optimization through global search and adaptive mechanisms. We chose the GWO framework as the baseline for developing FMGWO for several pragmatic and theoretical reasons. First, GWO is a population-based metaheuristic whose leadership hierarchy and hunting mechanism provide an intuitive balance between exploration and exploitation, which aligns well with the nature of continuous node-placement problems in WSN coverage. Second, the canonical GWO possesses a relatively small number of tunable parameters and a simple update scheme, which makes it straightforward to analyze and hybridize with auxiliary mechanisms. Third, the literature shows that GWO exhibits competitive performance on many continuous optimization benchmarks, and it has been successfully applied to placement and coverage problems; thus, it represents a suitable and well-understood starting point for targeted algorithmic improvements. At the same time, the standard GWO has known limitations—most notably a tendency toward premature convergence and diminished population diversity in later stages of optimization. These limitations motivate the targeted enhancements presented in this work, each designed to alleviate specific weaknesses without substantially complicating the algorithm’s structure. The findings of the present study propose the implementation of FMGWO, with the objective of achieving substantial enhancement in the coverage and resource efficiency of WSNs, thereby addressing the prevailing challenges in the context of practical applications.

## 3. Wireless Sensor Network Coverage Problem and Standard GWO

### 3.1. Sensor Network Node Coverage Model

In WSNs, the sensing capability of sensor nodes is a pivotal component in ensuring the efficacy of network monitoring. In this study, the sensing range of a node is defined as a circular area centered on the node with a sensing radius of R. It is demonstrated that only monitoring points within this area can be effectively sensed. The sensing radius, R, a pivotal parameter, directly determines the coverage efficiency of wireless sensor networks. In order to facilitate an analysis of complex problems, this study simplifies the WSN coverage area into a two-dimensional plane and develops the study based on the following idealized assumptions.

**Assumption** **1.** *The sensing range of all nodes is a perfect circular area and is not affected by any obstacles or environmental factors.*

**Assumption** **2.** *All sensor nodes have the same hardware structure and sensing capability to ensure a consistent sensing range.*

**Assumption** **3.** *All sensor nodes are mobile and can sense other nodes within their sensing range in real time and obtain the precise location information of these nodes.*

Based on the above assumptions, the WSN coverage model is as follows.

In this paper, the monitoring area is defined as a two-dimensional rectangular plane whose length is denoted as Y and whose width is denoted as Z, while the total area is Y × Z. In the planar rectangular coordinate system, the four vertices of this rectangular area have the following coordinates: (0, 0), (0, Z), (Y, 0), and (Y, Z). In the discretization process, the monitoring area is divided into n equal-area and symmetric grids. The center point of each grid is designated as the monitoring point, and its set is represented as follows.

(1) $$J=\left\{J_{j}=(x_{j},y_{j})|j=1,2,\ldots ,n\right\}$$

Next, v sensor nodes are randomly deployed in the monitoring area, the set of which is represented as follows.

(2) $$O=\{O_{i}=(x_{i},y_{i})|i=1,2,\ldots ,v\}$$

All nodes adhere to a Boolean perception model, with each node possessing a perception radius of R.

The calculation of the Euclidean distance between the sensor node, O_i_, and the monitoring point, J_j_, is achieved through the following equation.

(3) $$d(O_{i},J_{j})=\sqrt{{\left(x_{i}-x_{j}\right)}^{2}+{\left(y_{i}-y_{j}\right)}^{2}}$$

In Equation (3), $d(O_{i},J_{j})$ denotes the Euclidean distance between sensor node O_i_ and monitoring point J_j_. The Boolean perception function, P(O_i_,J_j_), is utilized to ascertain whether the sensor node is capable of detecting the target, the definition of which is as follows.

(4) $$P(O_{i},J_{j})=\left\{\begin{array}{ll} 1 & \mathrm{if}\;d(O_{i},J_{j})\leq R\\ 0 & \mathrm{otherwise} \end{array}\right.$$

In the event that this distance is less than or equal to the sensing radius, R, the monitoring point, J_j_, is within the sensing range of the sensor node, O_i_. This indicates that the grid where the monitoring point, J_j_, is located is covered by the WSN. As the same monitoring point, designated as J_j_, may be repeatedly sensed via multiple sensor nodes, the joint probability of sensing monitoring point J via all sensor nodes is given by the following formula.

(5) $$\eta (O_{\mathrm{all}},J_{j})=1-\prod_{i=1}^{v}(1-P(O_{i},J_{j}))$$

In Equation (5), $\eta (O_{\mathrm{all}},J_{j})$ denotes the joint probability that monitoring point J_j_ is sensed via at least one node in the monitoring area, O_all_ denotes all sensor nodes, and v is the total number of sensor nodes.

The evaluation of the performance of a WSN is contingent upon the utilization of an appropriate metric. One such metric is coverage, which is of paramount importance in this regard. In this model, the coverage ratio is defined as the ratio of the coverage area to the total area of the monitoring area. The coverage area is calculated by the product of the sum of the joint perception probabilities of all the monitoring points and the area of each grid. The calculation of the coverage ratio is as follows:

(6) $$C_{r}=\frac{\sum_{j=1}^{n}\eta (O_{\mathrm{all}},J_{j})\times \kappa }{Y\times Z}$$

(7) $$\kappa =\frac{Y\times Z}{n}$$

In Equations (6) and (7), C_r_ denotes the coverage ratio, Y × Z denotes the total area of the monitoring area, κ is the area of a single grid, and n is the number of grids. The coverage of the WSN is obtained by calculating the ratio of the area of the covered grid to the total area of the monitoring area.

### 3.2. Coverage Optimization Model

In order to achieve the optimal solution for the coverage performance of WSN in dynamic deployment, this study takes the coverage C_r_ as the optimization objective and constructs the corresponding objective function with the following mathematical expression.

(8) $$\begin{array}{l} \max{\;C}_{r}\\ s.t.\left\{\begin{array}{l} O_{i}\in G\\ J_{j}\in G \end{array}\right. \end{array}$$

In Equation (8), C_r_ denotes the coverage rate of the WSN, which is determined by Equation (6). O_i_ and J_j_ represent the coordinates of the i-th sensor node and the j-th monitoring point in the monitoring area, respectively, and G is the monitoring area.

The present study proposes a solution to the aforementioned optimization problem by introducing the GWO algorithm and embedding the objective function C_r_ into the fitness function of GWO. In the implementation of the algorithm, the monitoring area, G, is defined as the search space, and the coordinates of all v sensor nodes are mapped as dimension parameters of the gray wolf individuals. The first v dimensions correspond to the *x*-axis coordinates of all deployed nodes, and the last v dimensions correspond to their *y*-axis coordinates. The GWO iteratively optimizes the population positions through a high-frequency updating mechanism, and constraints within the search space ensure the feasibility of the solutions. Consequently, the optimal individuals resulting from the convergence of the algorithm correspond to the optimal node distribution scheme of the WSN, thereby maximizing the coverage. Note that the proposed FMGWO does not always guarantee finding the global optimal placement of sensor nodes, as it is a metaheuristic algorithm that approximates optimal solutions in complex, NP-hard problems like WSN coverage optimization. The optimal placement would ideally cover the rectangle with minimal overlap and no gaps, but due to the problem’s complexity, FMGWO provides near-optimal solutions, as validated through comparisons with other heuristics in the experiments.

### 3.3. Standard GWO

GWO is a metaheuristic optimization method inspired by the social behavior of grey wolves in nature. GWO’s design is based on the efficient simulation of the hierarchical structure of gray wolf packs with hunting strategies. The algorithm divides the pack into four hierarchical roles: alpha (α), beta (β), delta (δ), and omega (ω), which represent leaders, sub-leaders, secondary decision makers, and ordinary members of the pack, respectively. The wolves labeled α, β, and δ play the dominant roles in optimization and are responsible for guiding the direction of the search, whereas omega wolves adjust their behaviors based on the positional information of these dominant wolves. The wolves classified as α, β, and δ play a dominant role in the optimization process, guiding the search direction, while the wolves classified as ω adjust their behavior based on the position information of these dominant wolves.

The GWO algorithm is predicated on simulating the hunting behavior of gray wolves. This behavior can be divided into three phases: encircling the prey, tracking the prey, and executing the attack. The algorithm delineates the collaborative behavior of the wolf pack through a mathematical model and iteratively updates the positions of individuals step by step to approximate the global optimal solution. Within the search space, the position of each wolf individual denotes a potential solution. The following formula is employed to model hunting behavior in the GWO algorithm.

(9) $$X_{t+1}=X_{t}+A\cdot D\cdot X_{p}-A\cdot D\cdot X_{t}$$

In Equation (9), X_t+1_ denotes the location of the next generation of wolves, X_t_ denotes the location of the contemporary wolves, X_p_ denotes the location of the prey, and A and D denote the coefficients and distances, respectively.

(10) $$A=2\cdot a\cdot r_{1}-a$$

(11) $$C=2\cdot r_{2}$$

(12) $$a=2-2\cdot \frac{t}{T}$$

In the formula, C is the perturbation parameter for correcting the prey position, which is determined via Equation (13). r1 and r2 are random numbers between (0, 1), and a is the convergence factor, whose value decreases linearly from 2 to 0, which is calculated through Equation (14). t denotes the number of current iterations, and T denotes the total number of iterations.

In the GWO algorithm, the core of the leadership hierarchy is reflected in the role of wolves α, β, and δ in guiding the search direction of the entire wolf pack. Among them, wolf α, wolf β, and wolf δ represent the optimal solution, the second-best solution, and the third-best solution in the current iteration, respectively. During the iteration process, wolf ω dynamically adjusts its position based on the information provided by the three dominant wolves, and its trajectory is described by Equations (15) and (16). The following is the position update formula for the leadership level.

(13) $$\left\{\begin{array}{c} D_{\alpha }=\left|C_{1}\cdot X_{\alpha }-X_{t}\right|\\ D_{\beta }=\left|C_{2}\cdot X_{\beta }-X_{t}\right|\\ D_{\delta }=\left|C_{3}\cdot X_{\delta }-X_{t}\right| \end{array}\right.$$

(14) $$\left\{\begin{array}{c} X_{1}=X_{\alpha }-A_{1}\cdot D_{\alpha }\\ X_{2}=X_{\beta }-A_{2}\cdot D_{\beta }\\ X_{3}=X_{\delta }-A_{3}\cdot D_{\delta } \end{array}\right.$$

In Equations (13) and (14), D_α_, D_β_, and D_δ_ represent the distances between the specified gray wolf and wolves α, β, and δ, respectively. A_1_, A_2_, and A_3_ are the parameter control vectors that determine the direction of motion for the individual gray wolf. These vectors are calculated using the following Equation (11), and C_1_, C_2_, and C_3_ correct the ingested parameters for the positions of wolf α, wolf β, and wolf δ, which are computed in Equation (13). X_α_, X_β_, and X_δ_ are the wolf α, wolf β, and wolf δ position vectors, and X_1_, X_2_, and X_3_ are temporary positions.

(15) $$X_{t+1}=\frac{X_{1}+X_{2}+X_{3}}{3}$$

During the execution of the algorithm, the roles of α, β, and δ wolves are defined. Specifically, these wolves are responsible for recognizing the location of the prey and moving in the direction of the prey to pursue it. Concurrently, the three alpha wolves direct the other wolves to assume their positions, thereby facilitating the encirclement and capture of the prey through a series of coordinated actions. This collaborative effort ultimately culminates in the attainment of the optimal solution. The position update process of the wolf pack is governed by Equation (15). The time complexity of the standard GWO is O (N × D × T), where N is the population size, D is the problem dimension, and T is the number of iterations, primarily due to fitness evaluations and position updates in each iteration.

## 4. FMGWO

The standard GWO algorithm demonstrates considerable application potential in a variety of optimization problems, particularly in addressing the WSN coverage problem, exhibiting certain advantages over other swarm intelligence algorithms. However, its intrinsic mechanism still exhibits limitations in complex, high-dimensional, or multi-peak optimization scenarios. Specifically, GWO’s random initialization results in uneven node distributions, producing coverage gaps in WSNs. Its linear convergence factor lacks adaptability, hindering the balance between global exploration of diverse node layouts and local exploitation to minimize overlaps. The reliance on current alpha wolf information ignores historical high-quality node configurations, reducing robustness. The alpha wolf selection mechanism risks diversity loss, leading to convergence stagnation in multi-peak WSN landscapes. Additionally, GWO’s simplistic position update struggles to navigate the complex, multi-peak search space, often trapping the algorithm in local optima. These shortcomings collectively impair standard GWO’s performance in WSN coverage optimization. To address these limitations, this paper proposes an FMGWO that incorporates multiple strategies to effectively overcome the aforementioned shortcomings. The specific improvements are described as follows.

### 4.1. Electrostatic Field Initialization

To address the problem of the random distribution of nodes in WSNs tending to lead to coverage blindness, an electrostatic field model is used to generate a uniform initial population to improve the coverage efficiency. The standard GWO algorithm utilizes random generation during the initialization of the population. This approach is straightforward but may result in an imbalanced distribution of initial solutions within high-dimensional or intricate search spaces. This lack of initial diversity weakens global exploration, as nodes cluster in suboptimal regions, reducing the coverage rate. Consequently, a more scientific initialization method is required to enhance the diversity and coverage of the population.

**Figure 1:**
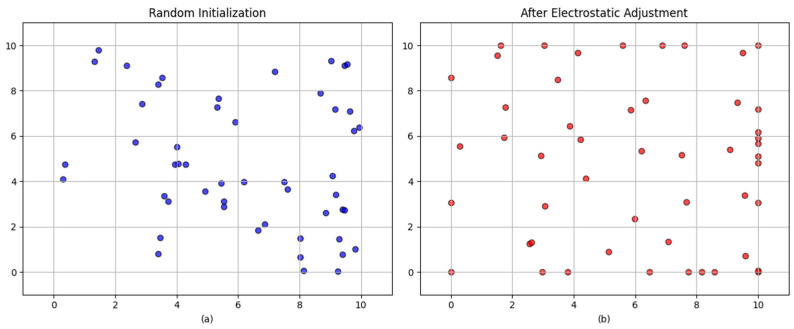
(**a**) Random initialization; (**b**) After electrostatic adjustment.

In order to address the issue of the imbalanced distribution of initial populations, this paper proposes an electrostatic field initialization strategy. The strategy under discussion draws inspiration from the theoretical framework of electrostatic fields as they apply in the physical sciences. In scenarios characterized by a low population number, the charged particles will tend to be uniformly distributed under the action of mutual repulsive force. By emulating the electrostatic force between particles, the initial population can be naturally dispersed in the search space, thereby providing a superior starting point for subsequent optimization. Furthermore, in comparison with the random initialization distribution, the electrostatic field initialization can better ensure enhanced stability during the initialization process. As illustrated in Figure 1, the initialization schematic following the application of the electrostatic field is presented (the population size is 50).

The electrostatic field initialization aims to produce an initial population that is more uniformly distributed across the search space and less likely to cluster prematurely. By using repulsive-like interactions during initialization, candidate solutions tend to avoid high-density clusters, which improves early exploration and provides better coverage of the solution manifold. The practical effect on coverage: more diverse initial placements yield a higher probability of finding well-distributed sensor configurations that cover sparse regions of the area. The fundamental principle underlying the initialization of the electrostatic field is the dynamic adjustment of the population’s position, a process determined by the calculation of distance and electrostatic force between individuals. First, the initial population location is randomly generated using a uniform distribution within the upper and lower bounds of the search space [L, U] with a scale of (N, m), where N denotes the population size and m denotes the problem dimension. For each pair of individuals, P_i_ and P_j_, in the population, the Euclidean distance, d, between them is computed. To avoid the potential division by zero error that can result from this calculation, a tiny value of 10^−6^ is introduced. This step ensures that the computation is stable even when the distance between individuals is extremely small. The formula is as follows.

(16) $$d=\max\left(\left\|P_{i}-P_{j}\right\|,10^{-6}\right)$$

Subsequently, in accordance with the inverse square law of electrostatic force, the magnitude of the force F is calculated. The force is directly proportional to the distance between the objects; therefore, as the distance between the objects decreases, the force of repulsion increases, causing the objects to be pushed apart. Furthermore, the direction vector of the force, σ, denoting the unit vector pointing from P_i_ to P_j_, must be calculated to ensure that the force acts along the line of connection between the individuals. The formula is as follows.

(17) $$F=\frac{1}{d^{2}}$$

(18) $$\sigma =\frac{P_{i}-P_{j}}{d}$$

In Equations (17) and (18), F denotes the magnitude of the electrostatic force, and σ denotes the direction vector of the force.

The individual positions are updated through the application of electrostatic forces, and a regulation factor, designated as k, is incorporated to govern the step size.

(19) $$P_{i}=P_{i}+k\cdot F\cdot \sigma$$

(20) $$P_{j}=P_{j}-k\cdot F\cdot \sigma$$

In Equation (20), P_i_ and P_j_ represent the positions of the ith and jth individuals in the population, respectively. These positions are assumed to move in opposite directions, thereby simulating the repulsion effect. The parameter k is the electrostatic force regulation coefficient, which serves to balance the magnitude of movement with stability.

Ultimately, the updated position is cropped to guarantee that the search space is not exceeded.

(21) $$P_{i}=\mathrm{clip}(P_{i},L,U)$$

In Equation (21), L denotes the upper bound of the search space and U denotes the lower bound of the search space.

This strategy is particularly effective for WSN coverage optimization, as it ensures a uniform initial node distribution, reducing coverage gaps in large-scale monitoring areas.

### 4.2. Dynamic Parameter Adjustment

In order to accommodate the constrained computational and energy resources of WSNs, this study adopts a balanced optimization strategy by integrating a nonlinear convergence factor with a differential evolution scaling parameter. The conventional GWO employs a linearly decreasing convergence factor (a), which monotonically decreases from 2 to 0 throughout the iterations. Although this approach offers simplicity, it exhibits inadequate adaptability when confronted with the complex and multimodal nature of WSN coverage optimization. A linear decay imposes a uniform reduction in search step size across all iterations, often resulting in excessive exploration in the early stages and insufficient exploitation toward the end of the search process. This rigidity impairs the algorithm’s ability to dynamically adjust its search behavior to problem-specific terrain, adversely affecting the convergence speed, precision, and the balance between exploration and exploitation [33].

In many biological systems, including the cooperative hunting of gray wolves, behavioral adjustments naturally exhibit nonlinear dynamics, where hunting speed, pursuit radius, and coordination strategies evolve according to environmental stimuli. Motivated by this observation, a nonlinear convergence factor is introduced to modulate the search radius more adaptively throughout the optimization process. At the early stage of iterations, the proposed nonlinear curve decreases more rapidly than its linear counterpart, enabling a swift contraction of the search range to intensify candidate solution refinement and accelerate convergence in promising regions. In the latter stage, the decay becomes gentler, preserving a certain degree of spatial exploration to reduce the probability of premature convergence to a local optimum. This nonlinear modulation effectively maintains a dynamic balance between intensification and diversification, thereby enhancing overall optimization accuracy, adaptability, and robustness in the presence of high-dimensional and irregular WSN topologies.

**Figure 2:**
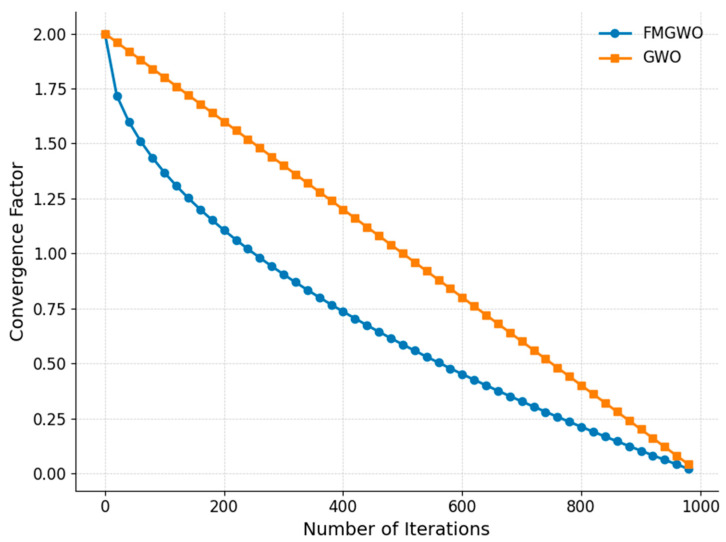
Convergence factor comparison curve.

From a theoretical standpoint, this adjustment alters the search dynamics by providing a variable exploration radius proportional to both iteration progress and the curvature of the nonlinear decay function. This facilitates a more problem-aware transition from exploration to exploitation, which is particularly beneficial for multimodal landscapes with irregular objective value distributions. Comparative results against the standard GWO (see Figure 2) demonstrate that the proposed nonlinear convergence factor yields a more desirable trade-off profile between the convergence rate and the solution quality. The nonlinear convergence factor (a) is formulated as follows.

(22) $$a=2\cdot \left(1-{\left(\frac{t}{T}\right)}^{0.5}\right)$$

In Equation (22), “a” denotes the convergence factor, “t” indicates the number of current iterations, and “T” signifies the maximum number of iterations.

Furthermore, the differential evolution scaling factor was recalibrated in this study. The magnitude of weighting in the differential evolution variant is controlled, thereby achieving a balance between global exploration and local exploitation.

(23) $$f(t)=0.5\left(1-\frac{t}{T}\right)$$

In Equation (23), the differential evolutionary scaling factor is denoted as f(t).

The mathematically nonlinear nature of the square root function enables the algorithm to adaptively adjust the search behavior. This adaptability renders the algorithm more flexible and suitable for complex optimization scenarios compared to linear decay.

By adaptively balancing exploration and exploitation, this approach addresses the dynamic and resource-constrained nature of WSNs, enabling efficient node placement.

### 4.3. Elder Council Mechanism

The standard GWO utilizes only the head wolf positions from the current iteration to guide the population update, with the alpha wolf serving as the principal leader in determining the search direction. However, high-quality solutions from earlier iterations, such as previous alpha positions with superior objective function values, are not preserved once replaced. This omission means that, if the current alpha is suboptimal, the algorithm is deprived of the ability to revert to historically superior solutions. As a result, the search process becomes more susceptible to premature convergence, potential quality degradation, and reduced robustness in WSN coverage optimization. In optimization theory, this represents a lack of temporal elitism retention, a concept well recognized in memory-based metaheuristics, where preservation of elite solutions can enhance both convergence stability and solution quality.

In order to address the problem of lost historical information and enhance the stability of the algorithm, the Council of Elders mechanism is proposed. The strategy draws inspiration from the elder system in human societies, wherein experienced elders are retained within the group to impart wisdom and guidance. In the context of wolf packs, this phenomenon can be conceptualized as analogous to the experience of the alpha wolf, who serves as the reference point for the pack. By periodically storing historical head wolf positions, the committee of elders provides a resource of candidate solutions for operations such as head wolf rotation, thereby avoiding the permanent loss of quality solutions.

The Council of Elders mechanism is implemented by periodically recording head wolf positions and limiting storage capacity. First, a storage set is created for alpha, beta, and delta, respectively, initially empty, for the purpose of recording historical positions. In each iteration, it is imperative to ascertain whether the current iteration number meets the update condition (i.e., every three generations). In the event that the specified condition is met, the current locations of alpha, beta, and delta are appended to the relevant storage collections.

(24) $$E_{a}=E_{a}\cup \{A\},\;E_{b}=E_{b}\cup \{B\},\;E_{d}=E_{d}\cup \{D\},\;\mathrm{if}\;t\;\mathrm{mod}\;3=0$$

In Equation (24), the variables E_a_, E_b_, and E_d_ represent a set of historical alpha, beta, and delta positions, respectively, which are stored in the Council of Elders. Initially, this set is empty. The variables A, B, and D correspond to the position vectors of the current alpha, beta, and delta, respectively. The variable t denotes the current iteration count.

In order to circumvent the accumulation of excessive historical data, the quantity of head wolves retained is constrained to a maximum of three per category. In the event that the limit is exceeded subsequent to the addition of new locations, the three most recent locations are retained, while the earliest records are removed, in order to ensure the currency of historical information.

(25) $$E_{x}=\left\{\begin{array}{ll} E_{x}, & \mathrm{if}\;|E_{x}|\leq 3\\ \{e_{i}|e_{i}\in E_{x},i>|E_{x}|-3\}, & \mathrm{if}\;|E_{x}|>3 \end{array}\right.,\;x\in \{\alpha ,\beta ,\delta \}$$

In Equation (25), x is the category identifier for elders, which takes values in the range of {α, β, δ} and corresponds to alpha, beta, and delta, respectively. |Ex| denotes the number of elements in the set Ex. e_i_ denotes the ith element in the set Ex, sorted in the order of additions. m denotes the problem dimension.

The Council of Elders’ primary function is to establish historical head wolf positions for the head wolf rotation mechanism as part of the Candidate Solution. They will not be directly involved in daily position updates.

This mechanism enhances the stability of WSN coverage optimization by preserving high-quality node layouts, preventing the loss of effective configurations in complex scenarios.

### 4.4. Alpha Wolf Tenure Inspection and Rotation

To mitigate premature convergence and preserve population diversity, this study introduces a tenure check and rotation mechanism for leader wolves in the GWO. In the standard GWO, the alpha, beta, and delta wolves are selected purely based on the instantaneous fitness ranking of the current population. While this procedure is computationally efficient, it cannot detect temporal stagnation; if the alpha wolf’s position remains unchanged or exhibits negligible fitness improvement across successive iterations, the search direction becomes overly dependent on a single, potentially suboptimal reference. This overreliance accelerates population clustering, reduces positional variance, and shrinks the effective exploration radius, often causing the algorithm to be trapped in a local optimum—a limitation particularly pronounced in WSN coverage optimization. Therefore, a strategy is required to detect and resolve this stagnant state.

This strategy draws inspiration from the phenomenon of leadership turnover observed among wolves in their natural habitat. In a wolf pack, when the incumbent leader is unable to effectively guide the pack forward due to advanced age or diminished capabilities, a new, strong individual typically assumes the leadership role. This turnover mechanism is instrumental in ensuring the continuity of the group’s adaptation to its environment. In a similar vein, the diversity of the algorithm and the capacity to circumvent local optima can be augmented by monitoring the performance of the alpha wolf and opting for a new alpha wolf from historical information and random perturbations when the need arises. The mechanism for monitoring and regulating the tenure of the alpha wolf is implemented through the use of counters and triggers. This mechanism is designed to initiate a rotation when a period of stagnation reaches a predetermined threshold.

First, a counter is initialized to keep track of the number of consecutive unimproved iterations of alpha adaptation. In each iteration, a comparison is made between the adaptation of the current alpha and the adaptation of the previous generation alpha. In the event that the counter remains unchanged, it is incremented; conversely, if an improvement is detected, the counter is reset to zero. This process quantifies the degree of stagnation of the alpha wolf.

(26) $$n_{t+1}=\left\{\begin{array}{ll} n_{t}+1, & \mathrm{if}\;S_{A}(t)=S_{L}\\ 0, & \mathrm{if}\;S_{A}(t)<S_{L} \end{array}\right.$$

(27) $$S_{L}=S_{A}(t),\;\mathrm{if}\;S_{A}(t)<S_{L}$$

As indicated in Equations (26) and (27), n_t_ is the counter value at the t-th iteration, denoting the number of consecutive unimproved generations of alpha scores, with an initial value of 0. $S_{A}(t)$ signifies the objective function score of alpha at the t-th iteration, while $S_{L}$ denotes the objective function score of alpha in the previous generation, with an initial value of ∞.

The rotation mechanism is activated once the counter reaches a preset threshold, which is set to five generations. The set of candidate solutions is to be constructed, including three types of sources: first, historical alpha, beta, and delta positions stored in the Council of Elders, which represent past quality solutions; second, alpha, beta, and delta positions of the current iteration, which retains the existing optimal solutions; and third, randomly perturbed solutions generated based on the current alpha positions, which use the normal distribution to introduce randomness to enhance the diversity.

(28) $$C=E_{a}\cup E_{b}\cup E_{d}\cup \{A,B,D\}\cup \{A+\lambda (0,a,m)\},\;\mathrm{if}\;n_{t}\geq 5$$

In Equation (28), C is the set of candidate solutions containing the historical head wolf, the current head wolf, and the randomly perturbed solutions. E_a_, E_b_, and E_d_ are the set of historical alpha, beta, and delta positions stored in the Council of Elders. A, B, and D are the position vectors of the current alpha, beta, and delta. λ(0,a,m) is a normally distributed stochastic vector with a mean of 0, a standard deviation of convergence factor of α, and a dimension of m.

The objective function value is computed for each solution in the set of candidate solutions, and the solution with the best fitness is selected as the new alpha.

(29) S={objf(p)∣p∈C}

(30) i=argmin{S}

(31) $$A=C\left[i\right]$$

(32) $$S_{A}=S[i]$$

As illustrated in Equations (29)–(32), S denotes the set of objective function scores for the candidate solutions. An element, p, in the set of candidate solutions, C, denotes a specific candidate position vector. The index, i, indicates the candidate solution with the optimal score. The position vector of the alpha, A, and the objective function score of the alpha, $S_{A}$, are also defined.

(33) $$n_{t}=0$$

(34) $$E_{a}=E_{b}=E_{d}=\emptyset$$

Once the rotation is complete, we reset the counters and empty the Council of Elders to ensure that the history is updated from the new alpha and to avoid old data interfering with subsequent optimizations.

This strategy mitigates the risk of local optima in WSN coverage problems, ensuring diverse node placements that maximize monitoring efficiency.

### 4.5. Hybrid Mutation Strategy

The position update of the standard GWO algorithm relies exclusively on the guidance of the head wolves (alpha, beta, delta) to compute the new position via a linear combination. This single strategy has limitations when facing multi-peak or high-dimensional optimization problems. Due to the lack of additional diversity generation mechanisms, the population tends to converge to the local optimum prematurely, leading to an insufficient search capability. Furthermore, the standard GWO’s simplistic position update mechanism struggles to navigate the multi-peak search space of WSN coverage optimization, often trapping the algorithm in local optima. Consequently, a more effective mutation strategy is required to promote population diversity and enhance global search capability.

In order to enhance the global search capability and the ability to jump out of local optima, this paper introduces a hybrid mutation strategy that combines population difference learning via differential evolution [34] and random perturbation through the Cauchy distribution [35]. The exploration and development capability of the algorithm is enhanced through multi-level variation.

The hybrid mutation strategy is realized via multi-stage position updating, which gradually enhances the diversity and adaptability of the population.

Initially, the position of each individual is calculated based on the position of the head wolf. The distances to alpha, beta, and delta were calculated independently for each individual, and the step size was adjusted via random coefficients. Ultimately, the average of the three was taken as the preliminary solution. This stage preserves the bootstrapping properties of GWO.

(35) $$\left\{\begin{array}{l} \Delta \alpha =|C_{1}P_{\alpha }-P_{i}(t)|\\ \Delta \beta =|C_{2}P_{\beta }-P_{i}(t)|\\ \Delta \delta =|C_{3}P_{\delta }-P_{i}(t)| \end{array}\right.$$

(36) $$\left\{\begin{array}{l} X_{1}=P_{\alpha }-H_{1}\Delta \alpha \\ X_{2}=P_{\beta }-H_{2}\Delta \beta \\ X_{3}=P_{\delta }-H_{3}\Delta \delta \end{array}\right.$$

(37) $$Q(t)=\frac{X_{1}+X_{2}+X_{3}}{3}$$

In Equations (35)–(37), Δα, Δβ, and Δδ represent the distance vectors between the gray wolf individuals and wolves α, β, and δ, respectively. C_1_, C_2_, and C_3_ are the ingestion parameters that correct the positions of wolves α, β, and δ. $P_{i}(t)$ is the current position vector of the i-th individual. H_1_, H_2_, and H_3_ are the parameter control vectors that determine the direction of movement for the gray wolf individuals. P_α_, P_β_, and P_δ_ denote the position vectors of wolf α, wolf β, and wolf δ, respectively. X1, X2, and X3 are the temporary position vectors. Q(t) is the initial new position vector of the i-th individual in the t-th iteration.

Based on the preliminary position vector, the differential evolution mechanism is introduced in the t-th iteration. Two different individuals are randomly selected from the population, and the difference in their position vectors is calculated, weighted by a dynamic scaling factor, and superimposed on the preliminary position vector to form a variant position vector. This step utilizes inter-population differences to enhance diversity.

(38) $$V(t)=Q(t)+f(t)\cdot (P_{k_{1}}(t)-P_{k_{2}}(t))$$

(39) $$\left\{\begin{array}{l} k_{1}\neq k_{2}\neq i\\ k_{1},k_{2}\in \{1,2,\ldots ,N\} \end{array}\right.$$

As indicated by Equations (38) and (39), V(t) denotes the variant position vector of the i-th individual in the t-th iteration, while f(t) signifies the differential evolutionary scaling factor in the t-th iteration, which is determined by Equation (23). The position vectors of two randomly selected individuals in the population in the t-th iteration are represented by $P_{k_{1}}(t)$ and $P_{k_{2}}(t)$, respectively. The random indexes k1 and k2 are defined as such.

In the t-th iteration, a random perturbation is applied to the variant position vector. This perturbation is based on the Cauchy distribution, and it is applied with a certain probability. The long-tailed nature of the Cauchy distribution generates larger jump steps, which facilitate the escape from local optima. The magnitude of the perturbation is associated with the range of the search space, thereby ensuring that the stochasticity remains moderate.

(40) $$V(t)=\left\{\begin{array}{ll} V(t)+W(t), & \mathrm{if}\;u<0.2\\ V(t), & \mathrm{otherwise} \end{array}\right.$$

(41) W(t)=Cauchy(0,1,m)·0.1(U−L)

In Equations (40) and (41), W(t) is defined as the Cauchy perturbation vector in the t-th iteration. μ is a uniformly distributed random number with values in the range of [0, 1], which is employed to regulate the Cauchy perturbation probability. Cauchy(0,1,m) is a standard Cauchy-distributed random vector with a mean of 0, a scale parameter of 1, and a dimensionality of m. L denotes the upper bound of the search space, and U denotes the lower bound of the search space.

Prior to the conclusion of the t-th iteration, boundary constraints are imposed on the variant position vector. The fitness of the variant position vector is then compared with that of the preliminary position vector. The superior one is selected as the final position vector for that individual in this iteration. This process is designed to ensure that the quality of the individual is improved or maintained at each iteration.

(42) V(t)=clip(V(t),L,U)

(43) $$P_{i}(t+1)=\left\{\begin{array}{ll} V(t), & \mathrm{if}\;f_{\mathrm{obj}}(V(t))<f_{\mathrm{obj}}(Q(t))\\ Q(t), & \mathrm{otherwise} \end{array}\right.$$

As indicated by Equations (42) and (43), $P_{i}(t+1)$ denotes the final updated position vector of the i-th individual in the t + 1st iteration. The objective function, $f_{\mathrm{obj}}$, is employed to assess the fitness of the solution.

The hybrid mutation strategy is tailored to the multi-peak nature of WSN coverage optimization, enabling the algorithm to explore diverse node arrangements and achieve higher coverage rates.

### 4.6. Algorithm Steps

**Figure 3:**
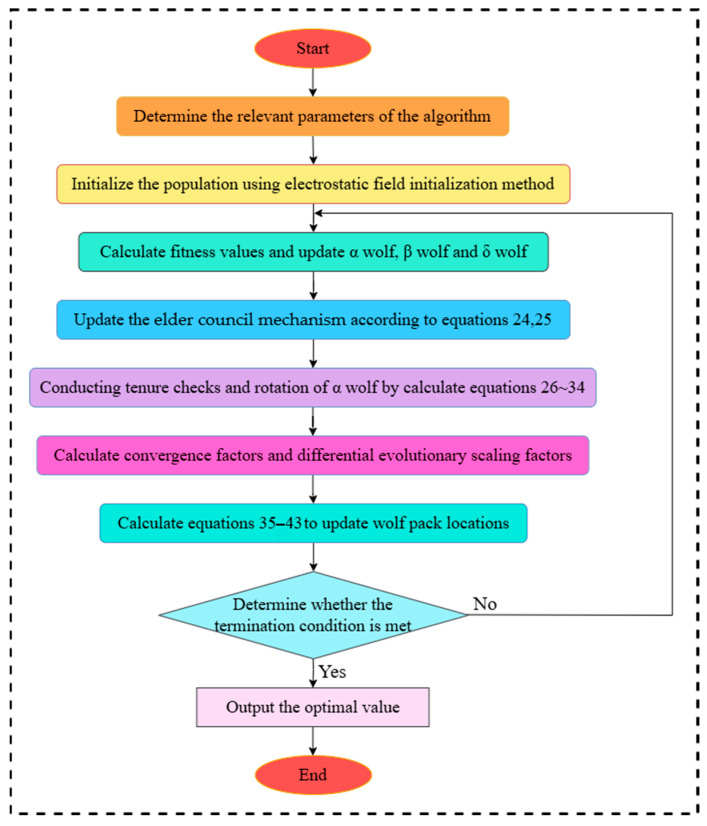
FMGWO algorithm flow chart.

Based on the above improvements, the implementation of the algorithm proposed in this paper can be divided into the following eight steps. As illustrated in Figure 3.

Step 1: determine the relevant parameters of the algorithm, including the population size, N, the maximum number of iterations T, and the search space.

Step 2: initialize the wolf pack and generate the initial position of the wolf pack in the search space using the electrostatic field initialization method.

Step 3: compute the fitness value and update wolf α, wolf β, and wolf δ.

Step 4: update the Council of Elders according to Equations (24) and (25).

Step 5: monitor the fitness of Wolf α and perform tenure checking and the rotation of Wolf α according to Equations (26)–(34).

Step 6: calculate the convergence factor and the differential evolutionary scaling factor.

Step 7: update the position of the mixed-variant strategy computed using Equations (35)–(43).

Step 8: Determine whether the current algorithm satisfies the optimal solution or the maximum number of iterations. If so, terminate the algorithm, and output the optimal solution. Otherwise, proceed to Step 3.

## 5. Experimental Design and Analysis

To verify the effectiveness of the algorithm improvement and its application performance in WSN coverage optimization, ablation experiments and application simulations using the algorithm were conducted. The experimental execution environment was an Intel (R) Core i9-12900H CPU with 2.90 GHz, 16 GB RAM, the Windows 11 64-bit operating system, and the Python 3.12 integrated development environment.

### 5.1. Design of Ablation Experiments

**Figure 4:**
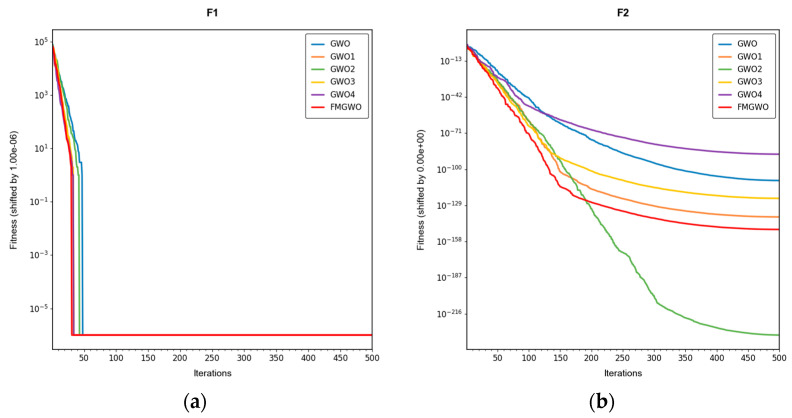

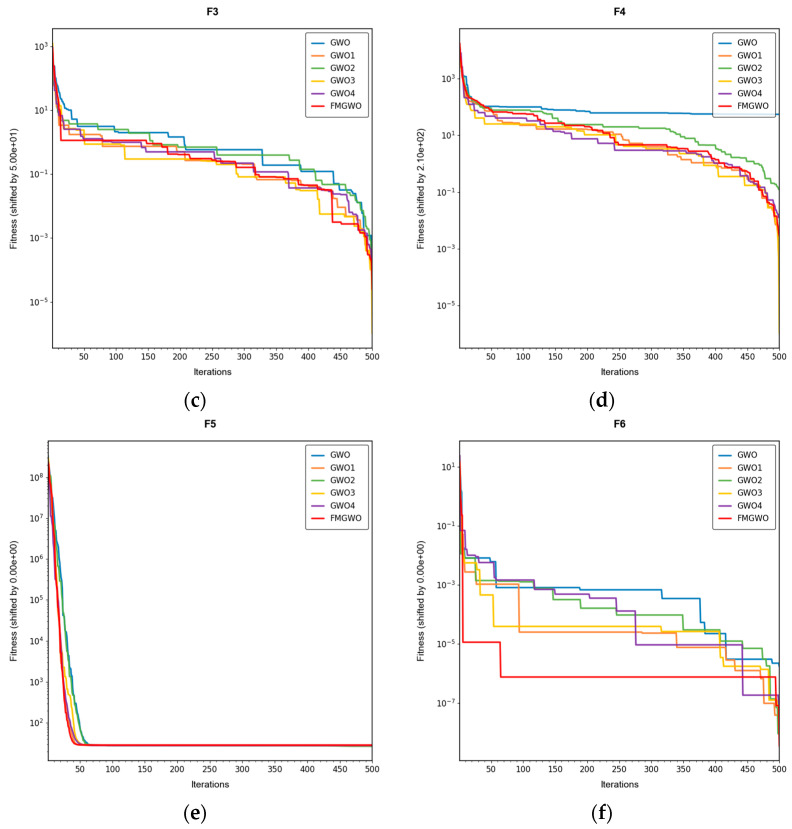
Convergence curves of basic unimodal functions, F1–F6.

**Figure 5:**
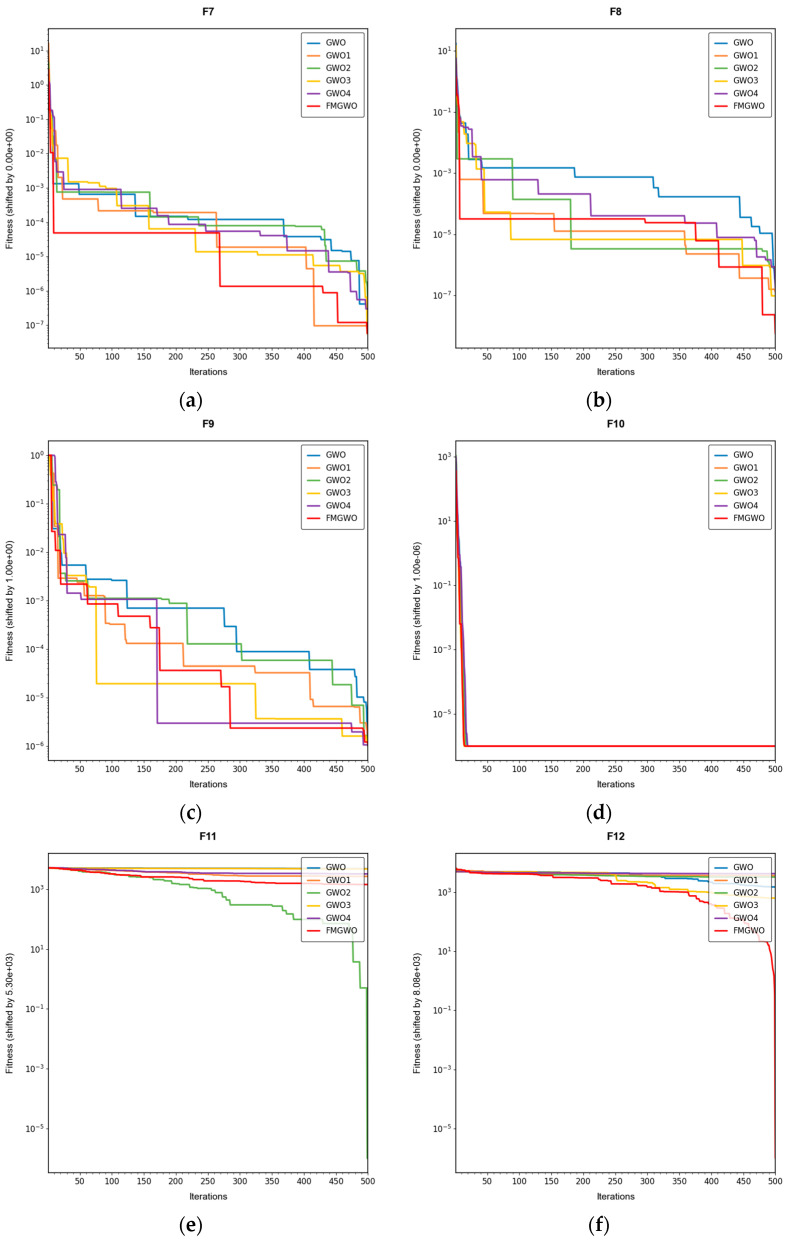

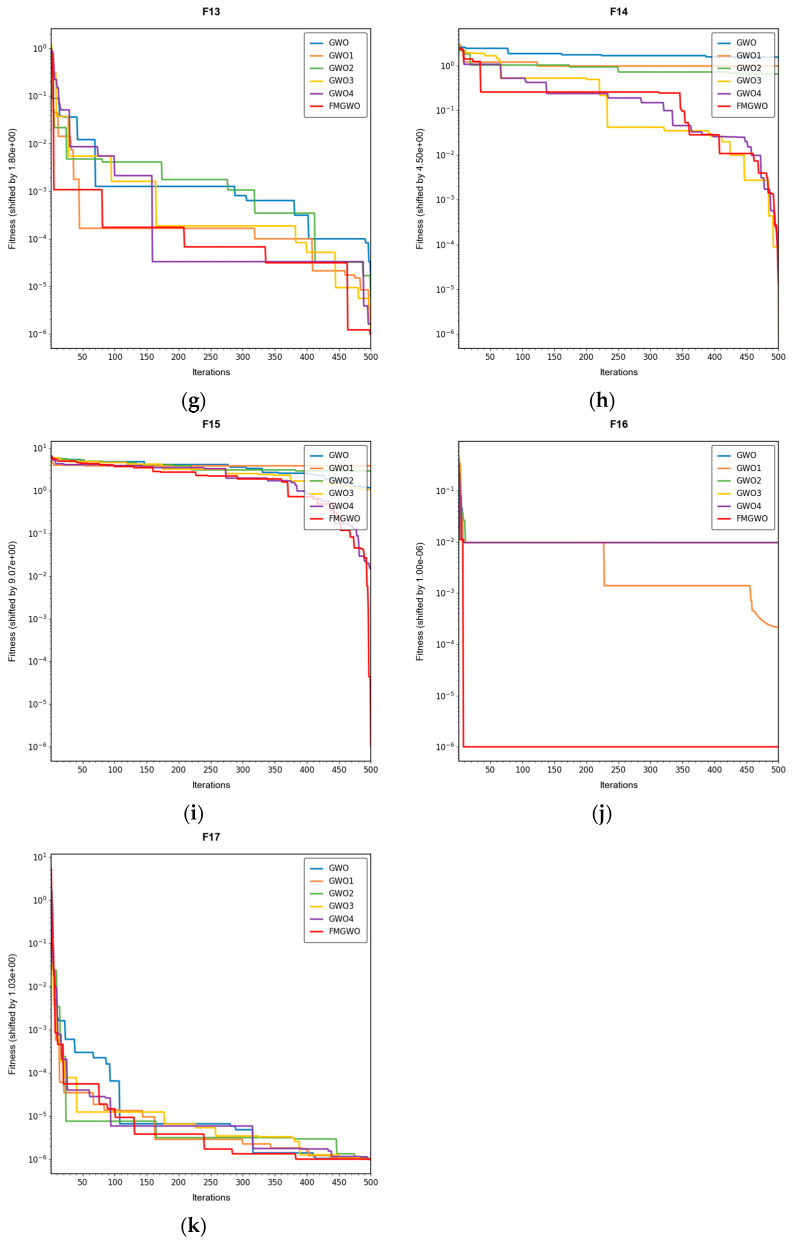
Convergence curves of classic multimodal functions, F7–F17.

**Figure 6:**
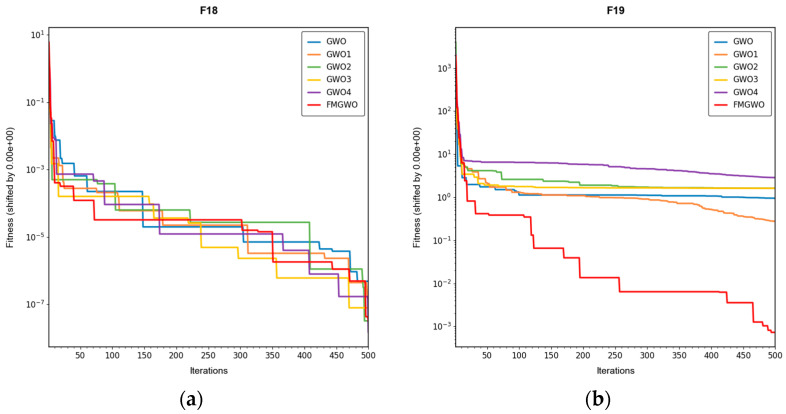

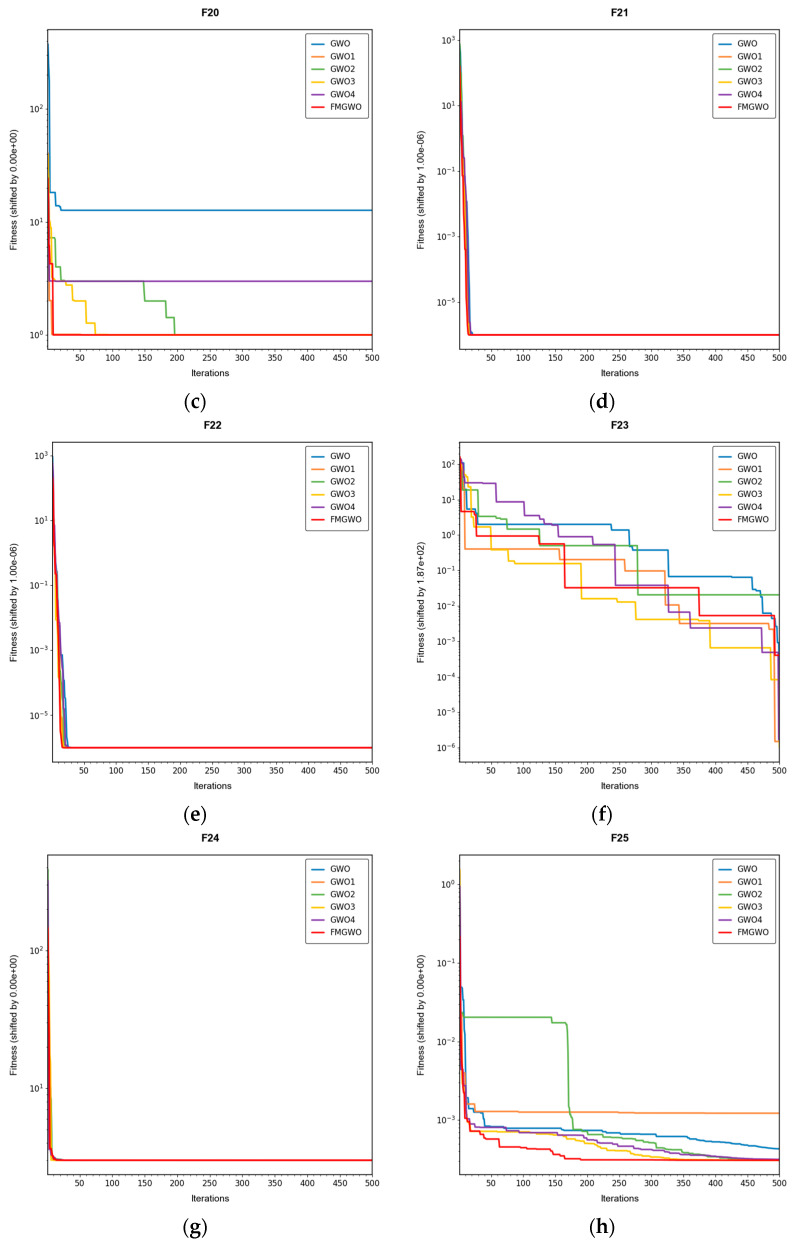

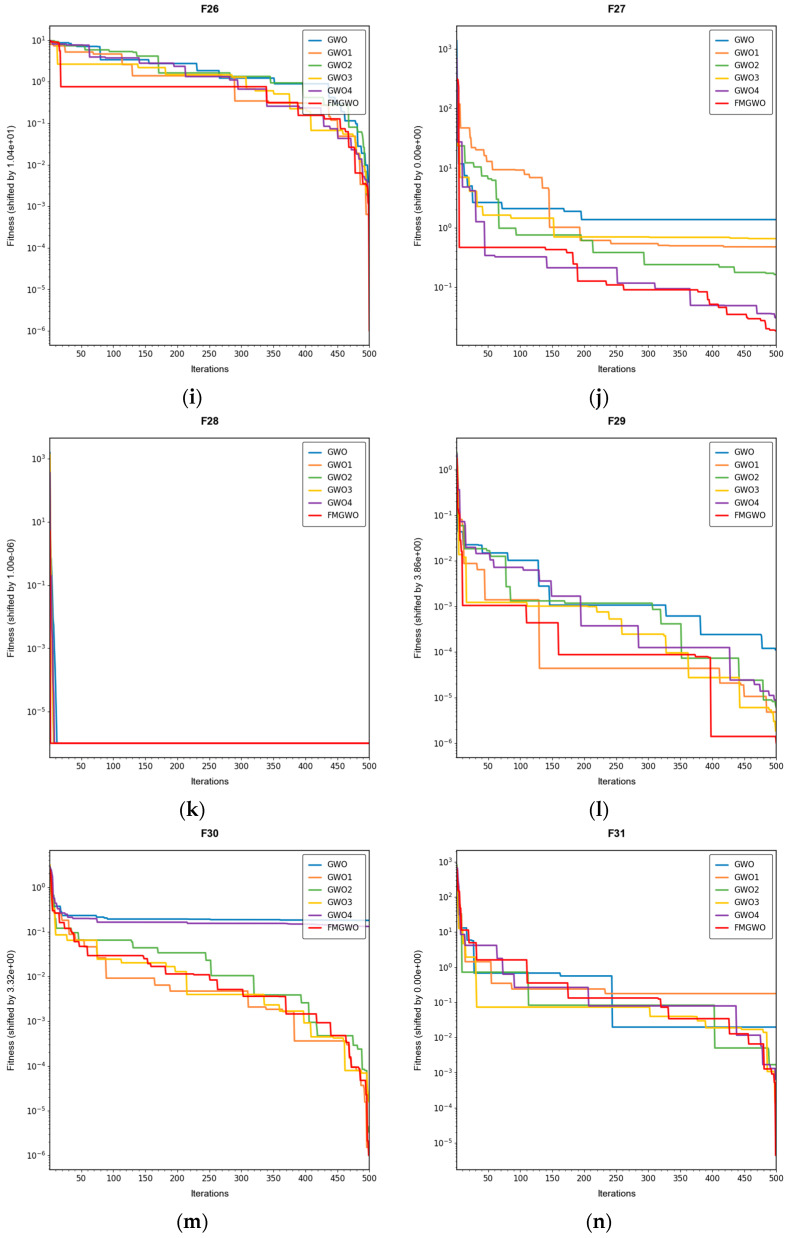

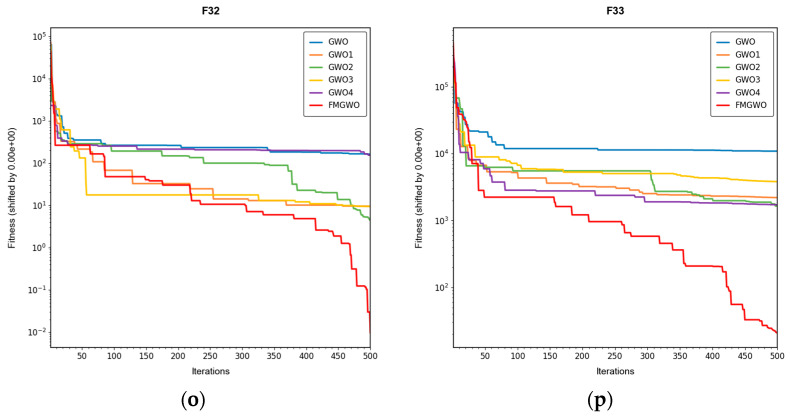
Convergence curves of complex composite functions, F18–F33.

To verify the effectiveness of the improved strategy, ablation experiments were performed using the improved FMGWO algorithm. The experimental comparison algorithm is shown in Table 1. A total of 33 benchmark functions were selected as test functions for the experiment in Table 2, Table 3 and Table 4. The selection of these functions was driven by their capacity to showcase a range of optimization challenges, encompassing high dimensionality, multi-modal, and intricate search spaces. These characteristics emulate those encountered in the domain of WSN coverage problems. The employment of FMGWO in the context of these functions serves two primary objectives. Firstly, it ensures the system’s resilience in confronting high-dimensional, non-linear optimization challenges. Secondly, it validates the system’s aptitude in addressing the specific challenges posed by the WSN coverage problem in a domain-specific manner. The population size of the algorithms was set to 30, and the number of iterations was set to 500. To ensure the stability of the experimental data, each algorithm was run independently 30 times, and the optimal, average, standard deviation, and worst values were taken as the performance comparison indices. The experimental results are shown in Table 5, Table 6, Table 7, Table 8, Table 9, Table 10, Table 11, Table 12, Table 13, Table 14, Table 15 and Table 16. The convergence curves are shown in Figure 4, Figure 5 and Figure 6.

In the course of analyzing the data for this study, it was discovered that certain data points were found to be in close proximity to a specific value during the data-processing and computational procedures. Consequently, these data points were directly approximated to that particular value when utilizing Excel formulas for computation. However, in reality, these data were not strictly equal to that particular value but were infinitely closer to it.

This phenomenon can be attributed to a confluence of factors, including the inherent characteristics of the data itself and the precision limitations inherent to Excel’s calculation capabilities. Excel employs distinct numerical precision and rounding rules when executing formula calculations. In instances where the discrepancy between the data and a specific value falls below Excel’s computational precision thresholds, the value is approximated as that specific value.

It is important to note that, although the data are shown as specific values in Excel, based on our in-depth understanding of the experimental process, data characteristics, and calculation methods, these data are actually infinitely close to that specific value rather than strictly equal. The data can be found with the standard deviation not being equal to zero. In the subsequent analysis of the data and discussion of the results, this characteristic was given full consideration. The data were processed and interpreted in a reasonable manner to ensure the accuracy and reliability of the research results.

When evaluating the convergence performance of the algorithms on functions, it should be noted that some of the fitness values may be non-positive. Consequently, the logarithmic scale cannot be applied directly for the visualization of these values. Consequently, a leveling operation is performed on a subset of the fitness values. It is important to note that this panning operation does not modify the relative disparity between the fitness values. Furthermore, it preserves the trend of the convergence curve, which can effectively demonstrate the iterative process of the algorithm. The shifted data are then employed to plot the convergence curve under the logarithmic scale, with the specific amount of shifting indicated in the graph description.

The experimental results demonstrate that the FMGWO algorithm exhibits superior performance in comparison to other algorithms when evaluated under a range of test functions, including basic unimodal functions, classic multimodal functions, and complex composite functions. These test functions are subsequently analyzed as follows:

All four strategies play a certain role in promoting the FMGWO algorithm.

The strategy in Section 4.1 enhances the initialization stage by combining a uniform distribution with controlled random perturbations. This ensures that the initial population is evenly dispersed across the search space, preventing local aggregation that often occurs in the standard GWO. By using a more balanced initialization, a sufficient number of candidate solutions are distributed across most feasible regions, laying a solid foundation for subsequent global exploration. Experimental results on unimodal functions F2 and F6 indicate that the standard GWO’s initial distribution can result in insufficient search coverage. When the Section 4.1 strategy is removed, GWO1 shows larger fluctuations across independent runs and, in several cases, fails to reach the global optimum. In contrast, FMGWO with the Section 4.1 strategy preserves higher diversity at the start of the search, avoids early local traps, and achieves better optimal and mean fitness values. This confirms that improving uniformity and stochasticity at initialization significantly enhances stability, the breadth of early exploration, and overall optimization robustness.

The strategy delineated in Section 4.2 is intended to enhance the algorithm’s capacity to conduct local searches with greater precision while ensuring that the depth and breadth of the global search are balanced. In practice, the search process often faces a trade-off between global exploration and local mining. It is easy to fall into a local optimum if only focusing on local search and difficult to converge if it is too decentralized. This strategy in FMGWO enables the algorithm to swiftly augment its advantage in the proximity of the optimal solution region by dynamically calibrating the search step size and implementing a local learning mechanism, while preserving a degree of jumping out capability in the global stage. An examination of the experimental data, such as the F6 test function, reveals that the standard GWO exhibits diminished local convergence performance. Additionally, a discernible discrepancy is evident between the optimal value and the mean value of GWO2 and FMGWO on specific functions. For instance, the optimal value of GWO2 in the F6 function is 1. In the context of the aforementioned numerical simulations, the convergence rate of 44 × 10^−7^ is observed to be less than that of FMGWO, which is found to decrease to 1.67 × 10^−8^. A similar trend is observed in the complex composite functions. While GWO2 performs marginally better than FMGWO in some of the tested functions, FMGWO ultimately demonstrates superior convergence speed and higher accuracy in most cases through this strategy. It can be argued that the Section 4.2 strategy effectively encourages individuals to seek out high-quality solutions within their local region. Additionally, its balancing mechanism serves to mitigate the loss of diversity that can result from too-fast convergence, thereby ensuring that the sensitivity of local search is not compromised while maintaining the effectiveness of a global search. A comprehensive analysis integrating the aforementioned mechanisms and data comparisons reveals that this strategy offers substantial advantages in enhancing solution accuracy and reducing convergence time, thereby playing a pivotal role in optimizing the overall performance of FMGWO.

Strategies 4.3 and 4.4 focus on addressing search stagnation by preserving high-quality solutions from historical iterations and integrating them into future searches. This prevents excessive reliance on the current best individual, thereby reducing the risk of local entrapment. The use of historical information gives the population a “memory effect,” guiding it toward unexplored yet promising regions. Comparative tests—particularly on F2, F11, and other functions—show that GWO3 consistently underperforms FMGWO, especially on unimodal, multimodal, and composite function benchmarks. Without these “memory” capabilities, the algorithm is prone to forgetting good solutions and repeatedly converging to suboptimal areas in complex landscapes. The combined effect of Section 4.3 and Section 4.4 is twofold: accelerating early search through historical guidance, and maintaining adaptability to escape local optima later, as reflected in smoother convergence curves. This long-term perspective greatly enhances the algorithm’s resilience and supports fine-tuning accuracy in challenging optimization tasks.

Section 4.5 introduces mutations and perturbations to maintain population diversity, particularly vital in high-dimensional, multi-peak problems. Without sufficient diversity, traditional algorithms tend to concentrate in a few local regions while neglecting potentially optimal areas. Data from composite functions F32 and F33 show that the standard GWO’s best values stagnate earlier at higher levels, and in some cases GWO4 even underperforms the original GWO. In contrast, FMGWO with Section 4.5 maintains wide coverage early through random perturbations, and it prevents over-convergence later in the search. This ensures the continuous exploration of new regions and improves global search performance, as confirmed by convergence curves showing a gradual, stable descent on multi-peak problems. Overall, this strategy significantly delays premature convergence, enhances population diversity, and provides the algorithm with a stronger global perspective in complex search spaces.

### 5.2. Wireless Sensor Network Coverage Experiment

The effectiveness of FMGWO in optimizing the WSN coverage problem is evaluated by using the coverage C_r_ (see Equation (6)) as the fitness value. The experimental parameters are listed in Table 17. Among them, PSO [36], GWO [27], CSA [37], DE [34], GA [38], FA [39], OGWO [40], DGWO1 [41], DGWO2 [41], and FMGWO optimize the coverage of WSNs, respectively. The superiority of FMGWO over other algorithms is verified by comparing the coverage C_r_ of each algorithm. The parameter settings of the above algorithms are shown in Table 18.

For practical implementation, we assume that each sensor node is equipped with localization capabilities to determine its exact position, as is common in many modern WSN applications. The computation of FMGWO is performed centrally at a sink node or base station that has sufficient computational resources and energy. Sensor nodes periodically report their positions to the sink via multi-hop communication, assuming a connected network. The sink then computes the optimized positions and broadcasts repositioning commands back to the nodes. This centralized approach minimizes the computational burden on energy-constrained sensor nodes, though it requires reliable communication links and assumes nodes have mobility capabilities for repositioning. These requirements introduce additional energy overhead for communication and movement, which should be considered in resource-limited deployments.

In the simulation setup, sensor nodes are initially deployed at random locations within the 100 m × 100 m monitoring area, as per the random deployment strategy described in Section 4.1. The algorithms then iteratively optimize node positions to maximize coverage, with performance examined through metrics such as best coverage, mean coverage, and standard deviation over 30 independent runs, ensuring statistical reliability.

These parameters were selected based on standard practices in the swarm intelligence literature for similar optimization problems: a population size of 30 balances exploration and computational efficiency, 500 iterations ensure sufficient convergence without excessive runtime, and algorithm-specific values follow original proposals or empirical tuning for WSN scenarios to promote effective search. In order to verify that each algorithm achieves the highest coverage with the minimum number of nodes, we conducted simulation experiments and selected three scenarios of 20, 25, and 30 nodes from small to large for comparison and analysis. Each comparison algorithm was run independently for 30 times, and the final coverage optimum, mean, and standard deviation were used as the comparison data. The experimental results are shown in Table 19.

The FMGWO algorithm demonstrates notable efficacy across a range of node counts. When the number of nodes is 20, the optimal coverage is 84.39%, and the average coverage is 82.47%. These values are superior to those obtained via other algorithms, such as PSO and GWO. The standard deviation of 0.0092 demonstrates the stability of the algorithm. As the number of nodes is increased from 25 to 30, the optimal coverage rate increases from 94.85% to 98.63%. Concurrently, the average coverage rate continues to exceed the other rates, while the standard deviation experiences a slight increase. However, the high coverage rate persists. A comparative analysis of the FMGWO algorithm with other algorithms reveals its superiority in key performance indicators such as optimal coverage, average coverage, and stability. This reflects its ability to adaptively reposition nodes, ensuring connectivity and coverage. Furthermore, the FMGWO algorithm demonstrates a capacity for achieving high coverage with a reduced number of nodes.

**Figure 7:**
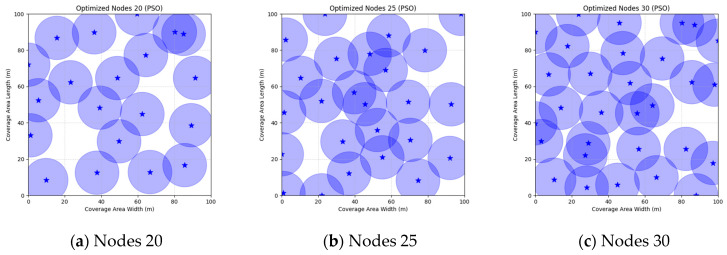
Optimizing WSN coverage with PSO.

**Figure 8:**
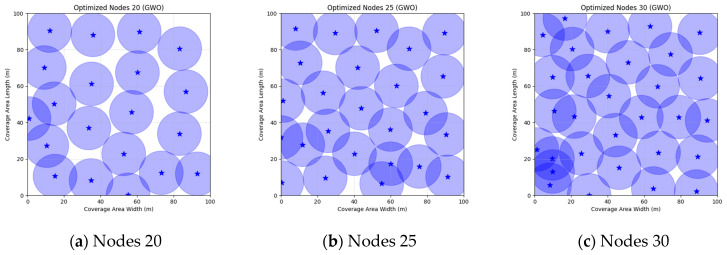
Optimizing WSN coverage with GWO.

**Figure 9:**
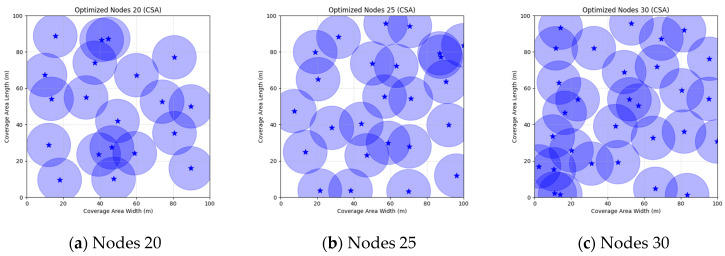
Optimizing WSN coverage with CSA.

**Figure 10:**
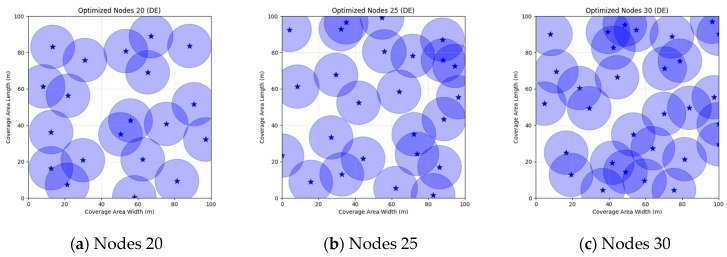
Optimizing WSN coverage with DE.

**Figure 11:**
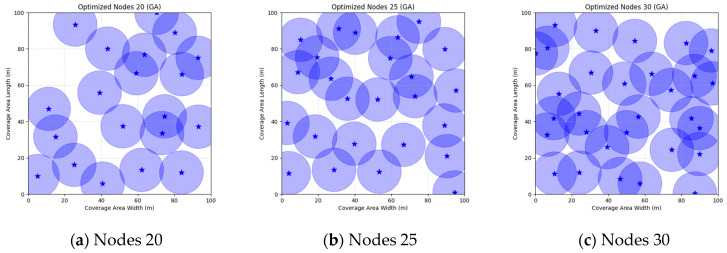
Optimizing WSN coverage with GA.

**Figure 12:**
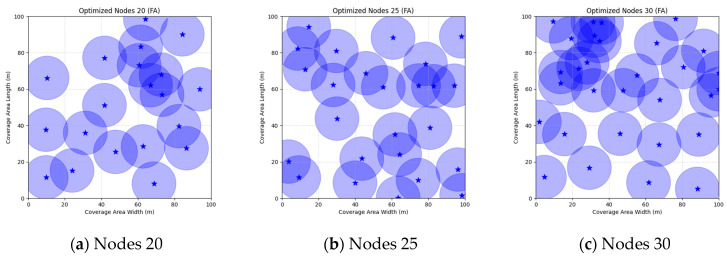
Optimizing WSN coverage with FA.

**Figure 13:**
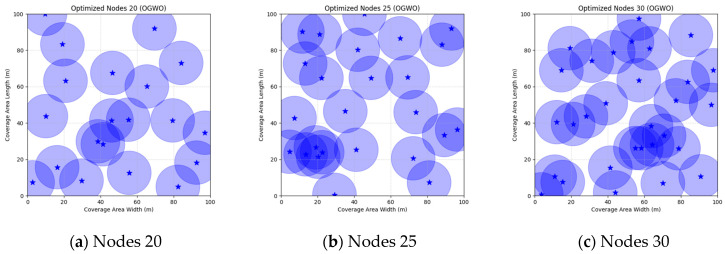
Optimizing WSN coverage with OGWO.

**Figure 14:**
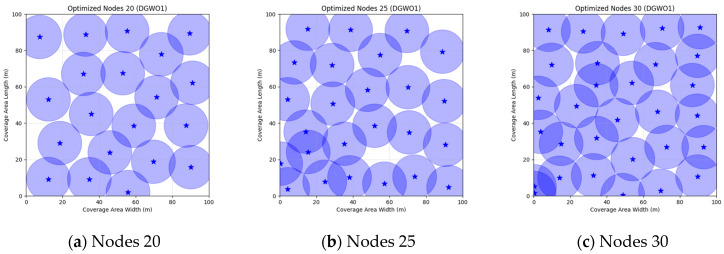
Optimizing WSN coverage with DGWO1.

**Figure 15:**
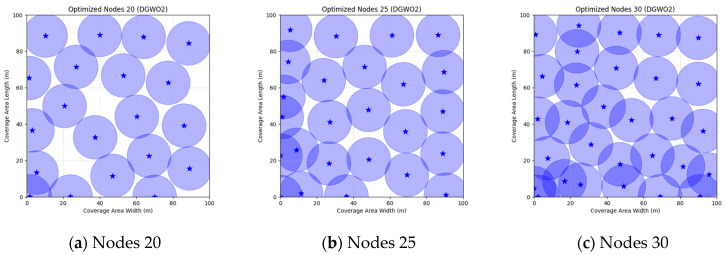
Optimizing WSN coverage with DGWO2.

**Figure 16:**
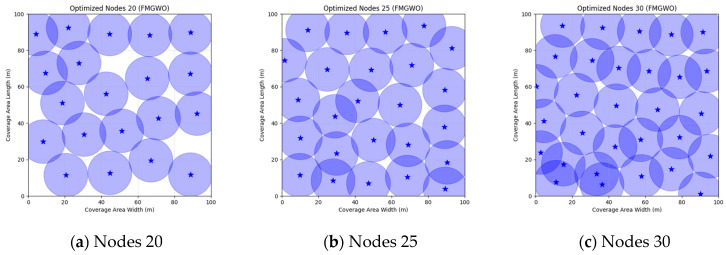
Optimizing WSN coverage with FMGWO.

A comparison of FMGWO with other algorithms reveals its superior performance. A comparison of the GWO with other algorithms, including PSO, GSA, FA, and GA, reveals that the GWO exhibits superior performance in terms of coverage rate across a range of node numbers. This observation suggests that the GWO enhances the standard GWO algorithm, thereby facilitating a more balanced exploration and development capacity and preventing the occurrence of local optimal solutions. When confronted with advanced algorithms such as OGWO, DGWO1, and DGWO2, FMGWO with 30 nodes exhibited an optimal coverage rate of 98.63% and an average coverage rate of 96.57%. These results substantiate the efficacy of the implemented improvement measures in enhancing the overall performance of the system. In practical applications, the FMGWO algorithm has been shown to achieve high coverage with fewer nodes, thereby effectively reducing costs. This has significant application value and broad application prospects. In order to provide a more intuitive demonstration of the efficacy of the FMGWO algorithm in addressing the WSN coverage problem, Figure 7, Figure 8, Figure 9, Figure 10, Figure 11, Figure 12, Figure 13, Figure 14, Figure 15 and Figure 16 illustrate the distribution of nodes associated with the optimization of the WSN coverage problem by each comparative algorithm. The asterisks (*) in the figure represent the specific locations of the sensor nodes, and the circular area clearly depicts the actual sensing range of each sensor node.

As demonstrated in Figure 7, Figure 8, Figure 9, Figure 10, Figure 11, Figure 12, Figure 13, Figure 14, Figure 15 and Figure 16, the FMGWO algorithm exhibits superior performance in comparison to the PSO, GWO, CSA, DE, GA, FA, OGWO, DGWO1, and DGWO2 algorithms when the number of sensor nodes is 20, 25, and 30, respectively. It has been demonstrated that these comparative algorithms are generally afflicted with two main issues. Firstly, there is an absence of comprehensive coverage of monitoring areas. Secondly, and perhaps more significantly, there is redundant monitoring in areas that have undergone optimization. Conversely, the FMGWO algorithm employs optimization to achieve a more uniform distribution of nodes, thereby reducing the redundant monitoring area and enhancing network coverage.

**Figure 17:**
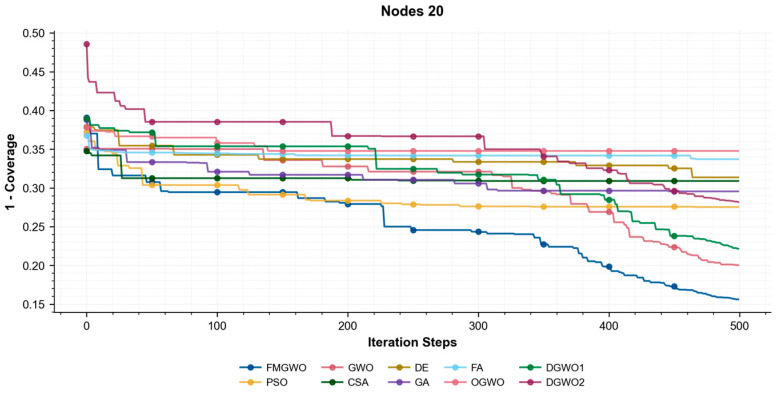
Convergence curves of each algorithm (Nodes 20).

**Figure 18:**
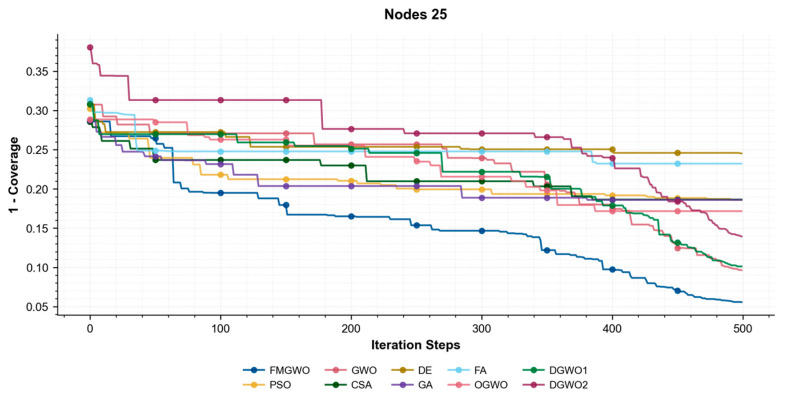
Convergence curves of each algorithm (Nodes 25).

**Figure 19:**
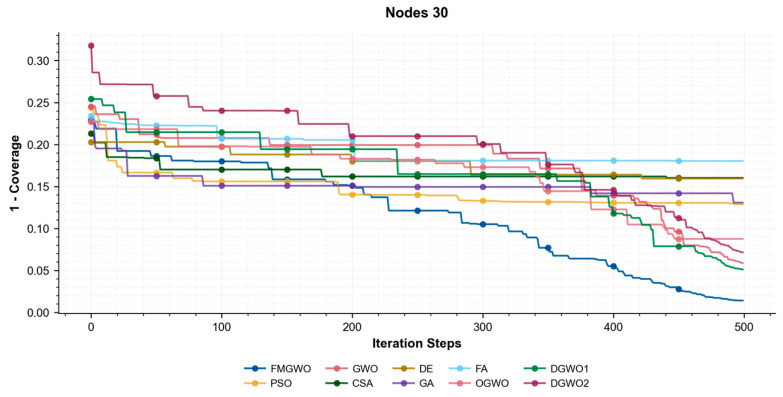
Convergence curves of each algorithm (Nodes 30).

As illustrated in Figure 17, Figure 18 and Figure 19, the coverage convergence curves for each algorithm are demonstrated after 500 iterations for three cases of 20, 25, and 30 nodes. The convergence accuracy and speed of the algorithms can be used to further understand their performance in optimizing the WSN coverage problem.

As demonstrated in Figure 17, Figure 18 and Figure 19, the FMGWO enhances the pre-convergence speed and post-convergence accuracy of WSN optimization while preserving the benefits inherent to GWO. The curves stabilize after approximately 450–500 iterations, as evidenced by the flattening of fitness values, indicating effective convergence to near-optimal solutions. The other two improved GWO algorithms mentioned above, i.e., OGWO, DGWO1, and DGWO2, are not as effective as FMGWO in optimizing the WSN coverage, although they improve GWO. Therefore, compared with the comparison algorithms, the FMGWO algorithm achieves stronger overall competitiveness in terms of searching accuracy, convergence speed, and stability.

## 6. Conclusions

In order to achieve the maximum coverage of WSN, this paper has proposed an improved gray wolf optimization algorithm (FMGWO). The FMGWO algorithm incorporates electrostatic field initialization, dynamic parameter tuning, an elder council mechanism, a head wolf tenure-checking and rotation mechanism, and a hybrid mutation strategy. The efficacy of the proposed strategies was initially validated through a series of ablation experiments, which demonstrate that these strategies enhance the accuracy and stability of the algorithm. Furthermore, the findings of simulation experiments demonstrate that the FMGWO algorithm possesses a distinct advantage in addressing the WSN coverage problem. It can be seen that FMGWO offers certain advantages in solving the WSN coverage problem, which is reflected in the fact that it not only effectively improves the coverage quality of network nodes but also achieves good stability. In summary, FMGWO excels in dynamic WSN deployment, offering a practical solution for real-time optimization under mobility and communication constraints. It helps maximize the network monitoring capability under limited resource conditions, which can effectively improve the network coverage efficiency and reduce the waste of resources in practical application scenarios. This is of great significance for WSN deployment.

## Figures and Tables

**Table 1:** Comparison algorithm name for ablation experiment.

Algorithm Name	Function Description
GWO	Standard gray wolf algorithm
GWO1	Only remove the strategy in Section 4.1 of this article
GWO2	Only remove the strategy in Section 4.2 of this article
GWO3	Only remove the strategy in Section 4.3 and Section 4.4 of this article
GWO4	Only remove the strategy in Section 4.5 of this article
FMGWO	Combining all strategies in this article

**Table 2:** Basic unimodal functions, F1–F6.

Function	n	Range	f_min_
$F1=\sum_{i=1}^{n}{\left(\left[x_{i}+0.5\right]\right)}^{2}$	30	[−100, 100]	0
$F2=0.26(x_{1}^{2}+x_{2}^{2})-0.48x_{1}x_{2}$	2	[−10, 10]	0
$F3=\sum_{i=1}^{n}{\left(x_{i}-1\right)}^{2}+\sum_{i=2}^{n}x_{i}x_{i-1}$	6	[−36, 36]	−50
$F4=\sum_{i=1}^{n}{\left(x_{i}-1\right)}^{2}+\sum_{i=2}^{n}x_{i}x_{i-1}$	10	[−100, 100]	−210
$F5=\sum_{i=1}^{n-1}\left(100{\left(x_{i+1}-x_{i}\right)}^{2}\right)+{\left(x_{i}-1\right)}^{2}$	30	[−30, 30]	0
$F6={\left(x_{1}+2x_{2}-7\right)}^{2}+{\left(2x_{1}+x_{2}-5\right)}^{2}$	2	[−10, 10]	0

**Table 3:** Classic multimodal functions, F7–F17.

Function	n	Range	f_min_
$\begin{array}{l} F7=\sin^{2}(3\pi x_{1})+{\left(x_{1}-1\right)}^{2}[1+\sin^{2}(3\pi x_{2})]\\ \;+{\left(x_{2}-1\right)}^{2}[1+\sin^{2}(2\pi x_{2})] \end{array}$	2	[−10, 10]	0
$\begin{array}{ll} F8= & \sin^{2}(\pi w_{1})+\sum_{i=1}^{n-1}{\left(w_{i}-1\right)}^{2}[1+10\sin^{2}(\pi w_{i}+1)]\\ & +{\left(w_{n}-1\right)}^{2}[1+\sin^{2}(2\pi w_{n})],\\ & w_{i}=1+\frac{x_{i}-1}{4},i=1,2,\ldots n \end{array}$	30	[−10, 10]	0
$F9=-cos(x_{1})cos(x_{2})e^{-{\left(x_{1}-2\right)}^{2}-{\left(x_{2}-\pi \right)}^{2}}$	2	[−100, 100]	−1
$F10=x_{1}^{2}+2x_{2}^{2}-0.3\cos(3\pi x_{1})-0.4\cos(4\pi x_{2})+0.7$	2	[−100, 100]	0
$F11=-\sum_{i=1}^{n}\left[x_{i}^{2}-10\cos(2\pi x_{i})+10\right]$	30	[−5.12, 5.12]	0
$F12=-\sum_{i=1}^{n}\left(x_{i}\sin(\sqrt{\left|x_{i}\right|})\right)$	30	[−500, 500]	−12,569.5
$F13=-\sum_{i=1}^{n}\sin(x_{i}){\left(\sin(ix_{i}^{2}/\pi )\right)}^{20}$	2	[0, π]	−1.8013
$F14=-\sum_{i=1}^{n}\sin(x_{i}){\left(\sin(ix_{i}^{2}/\pi )\right)}^{20}$	5	[0, π]	−4.6877
$F15=-\sum_{i=1}^{n}\sin(x_{i}){\left(\sin(ix_{i}^{2}/\pi )\right)}^{20}$	10	[0, π]	−9.6602
$F16=0.5+\frac{\sin^{2}(\sqrt{x_{1}^{2}+x_{2}^{2}})-0.5}{{\left(1+0.001(x_{1}^{2}+x_{2}^{2})\right)}^{2}}$	2	[−100, 100]	0
$F17=4x_{1}^{2}-2.1x_{1}^{4}+\frac{1}{3}x_{1}^{6}+x_{1}x_{2}-4x_{2}^{2}+4x_{2}^{4}$	2	[−5, 5]	−1.03163

**Table 4:** Complex composite functions, F18–F33.

Function	n	Range	f_min_
$\begin{array}{ll} F18 & ={\left(1.5-x_{1}+x_{1}x_{2}\right)}^{2}+{\left(2.25-x_{1}+x_{1}x_{2}^{2}\right)}^{2}\\ & +{\left(2.625-x_{1}+x_{1}x_{2}^{3}\right)}^{2} \end{array}$	5	[−4.5, 4.5]	0
$\begin{array}{ll} F19 & =100{\left(x_{1}-x_{2}\right)}^{2}+{\left(x_{1}-1\right)}^{2}+{\left(x_{4}-1\right)}^{2}+90{\left(x_{3}^{2}-x_{4}^{2}\right)}^{2}\\ & +10.1({\left(x_{2}-1\right)}^{2}+{\left(x_{4}-1\right)}^{2})+19.8(x_{2}-1)(x_{4}-1) \end{array}$	4	[−10, 10]	0
$F20={\left[\frac{1}{500}+\sum_{j=1}^{25}\frac{1}{j+\sum_{i=1}^{2}{\left(x_{i}-a_{ij}\right)}^{6}}\right]}^{-1}$	2	[−65.536, 65.536]	0.998
$F21=x_{1}^{2}+2x_{2}^{2}-0.3\cos(3\pi x_{1})(4\pi x_{3})+0.3$	2	[−100, 100]	0
$F22=x_{1}^{2}+2x_{2}^{2}-0.3\cos(3\pi x_{1}+4\pi x_{3})+0.3$	2	[−100, 100]	0
$F23=\left(\sum_{i=1}^{5}i\cos((i+1)x_{1}+i)\right)\left(\sum_{i=1}^{5}i\cos((i+1)x_{2}+i)\right)$	2	[−10, 10]	−186.7309
$\begin{array}{ll} F24= & \left[\begin{array}{l} 1+{\left(x_{1}+x_{2}+1\right)}^{2}(19-14x_{1}+3x_{1}^{2}\\ -14x_{2}+6x_{1}x_{2}+3x_{2}^{2}) \end{array}\right]\\ & \times \left[\begin{array}{l} 30+{\left(2x_{1}+1-3x_{2}\right)}^{2}(18-32x_{1}\\ +12x_{1}^{2}+48x_{2}-36x_{1}x_{2}+27x_{2}^{2}) \end{array}\right] \end{array}$	2	[−2, 2]	3
$F25=\sum_{i=1}^{11}{\left|a_{i}-\frac{x_{1}(b_{i}^{2}+b_{i}x_{2})}{b_{i}^{2}+b_{i}x_{3}+x_{4}}\right|}^{2}$	4	[−5, 5]	0.00031
$F26=-\sum_{i=1}^{10}{\left|(x_{i}-a_{i}){\left(x_{i}-a_{i}\right)}^{T}+c_{i}\right|}^{-1}$	4	[0, 10]	−10.4028
$F27=\sum_{k=1}^{n}{\left(\sum_{i=1}^{n}\left(i^{k}+\beta \right)\left({\left(\frac{x_{i}}{i}\right)}^{k}-1\right)\right)}^{2}$	4	[−4, 4]	0
$F28=\sum_{k=1}^{n}{\left(\left(\sum_{i=1}^{n}x_{i}^{k}\right)-b_{k}\right)}^{2}$	4	[0, 4]	0
$F29=-\sum_{i=1}^{4}\exp\left[-\sum_{j=1}^{3}a_{ij}{\left(x_{j}-p_{ij}\right)}^{2}\right]$	3	[0, 1]	−3.86
$F30=-\sum_{i=1}^{4}\exp\left[-\sum_{j=1}^{3}a_{ij}{\left(x_{j}-p_{ij}\right)}^{2}\right]$	6	[0, 1]	−3.32
$F31={\sum_{i=1}^{n}\left[\begin{array}{l} \sum_{j=1}^{n}\left(a_{ij}\sin\alpha_{j}+b_{ij}\cos\alpha_{j}\right)\\ -\sum_{j=1}^{n}\left(a_{ij}\sin x_{j}+b_{ij}\cos x_{j}\right) \end{array}\right]}^{2}$	2	[−π, π]	0
$F32={\sum_{i=1}^{n}\left[\begin{array}{l} \sum_{j=1}^{n}\left(a_{ij}\sin\alpha_{j}+b_{ij}\cos\alpha_{j}\right)\\ -\sum_{j=1}^{n}\left(a_{ij}\sin x_{j}+b_{ij}\cos x_{j}\right) \end{array}\right]}^{2}$	5	[−π, π]	0
$F33={\sum_{i=1}^{n}\left[\begin{array}{l} \sum_{j=1}^{n}\left(a_{ij}\sin\alpha_{j}+b_{ij}\cos\alpha_{j}\right)\\ -\sum_{j=1}^{n}\left(a_{ij}\sin x_{j}+b_{ij}\cos x_{j}\right) \end{array}\right]}^{2}$	10	[−π, π]	0

**Table 5:** Optimal values of each algorithm tested on F1–F6 functions.

Function	GWO	GWO1	GWO2	GWO3	GWO4	FMGWO
1	0.00	0.00	0.00	0.00	0.00	0.00
2	1.49 × 10^−121^	2.00 × 10^−147^	2.90 × 10^−236^	3.70 × 10^−144^	0.00	1.30 × 10^−153^
3	−5.00 × 10^1^	−5.00 × 10^1^	−5.00 × 10^1^	−5.00 × 10^1^	−5.00 × 10^1^	−5.00 × 10^1^
4	−2.10 × 10^2^	−2.10 × 10^2^	−2.10 × 10^2^	−2.10 × 10^2^	−2.10 × 10^2^	−2.10 × 10^2^
5	2.60 × 10^1^	2.60 × 10^1^	2.61 × 10^1^	2.53 × 10^1^	2.58 × 10^1^	2.56 × 10^1^
6	5.79 × 10^−9^	1.11 × 10^−8^	7.75 × 10^−8^	6.82 × 10^−9^	1.44 × 10^−8^	3.39 × 10^−9^

**Table 6:** Average of each algorithm tested on F1–F6 functions.

Function	GWO	GWO1	GWO2	GWO3	GWO4	FMGWO
1	0.00	0.00	0.00	0.00	0.00	0.00
2	2.30 × 10^−100^	9.60 × 10^−114^	5.40 × 10^−228^	1.70 × 10^−116^	1.02 × 10^−72^	1.00 × 10^−134^
3	−4.93 × 10^1^	−5.00 × 10^1^	−5.00 × 10^1^	−5.00 × 10^1^	−5.00 × 10^1^	−5.00 × 10^1^
4	−1.69 × 10^2^	−2.10 × 10^2^	−2.10 × 10^2^	−1.75 × 10^2^	−2.10 × 10^2^	−2.10 × 10^2^
5	2.71 × 10^1^	2.71 × 10^1^	2.62 × 10^1^	2.70 × 10^1^	2.78 × 10^1^	2.60 × 10^1^
6	7.07 × 10^−7^	1.21 × 10^−7^	1.44 × 10^−7^	8.22 × 10^−8^	1.21 × 10^−7^	1.67 × 10^−8^

**Table 7:** Standard deviation of each algorithm tested on F1–F6 functions.

Function	GWO	GWO1	GWO2	GWO3	GWO4	FMGWO
1	0.00	0.00	0.00	0.00	0.00	0.00
2	1.24 × 10^−99^	4.80 × 10^−113^	0.00	5.50 × 10^−116^	5.46 × 10^−72^	3.10 × 10^−134^
3	3.76	5.34 × 10^−5^	2.17 × 10^−5^	6.47 × 10^−5^	3.08 × 10^−5^	1.23 × 10^−5^
4	4.68 × 10^1^	1.24 × 10^−2^	3.31 × 10^−3^	5.56 × 10^1^	1.18 × 10^−2^	1.07 × 10^−3^
5	6.40 × 10^−1^	1.00	3.88 × 10^−2^	1.01	9.11 × 10^−1^	1.59 × 10^−1^
6	7.30 × 10^−7^	1.27 × 10^−7^	4.06 × 10^−8^	7.48 × 10^−8^	1.02 × 10^−7^	8.80 × 10^−9^

**Table 8:** Worst values of each algorithm tested on F1–F6 functions.

Function	GWO	GWO1	GWO2	GWO3	GWO4	FMGWO
1	0.00	0.00	0.00	0.00	0.00	0.00
2	6.91 × 10^−99^	2.70 × 10^−112^	5.60 × 10^−227^	2.90 × 10^−115^	3.04 × 10^−71^	1.40 × 10^−133^
3	−2.91 × 10^1^	−5.00 × 10^1^	−5.00 × 10^1^	−5.00 × 10^1^	−5.00 × 10^1^	−5.00 × 10^1^
4	−5.58 × 10^1^	−2.10 × 10^2^	−2.10 × 10^2^	−3.02 × 10^1^	−2.10 × 10^2^	−2.10 × 10^2^
5	2.80 × 10^1^	2.95 × 10^1^	2.62 × 10^1^	2.88 × 10^1^	2.91 × 10^1^	2.62 × 10^1^
6	3.04 × 10^−6^	5.03 × 10^−7^	2.23 × 10^−7^	3.61 × 10^−7^	4.34 × 10^−7^	3.21 × 10^−8^

**Table 9:** Optimal values of each algorithm tested on F7–F17 functions.

Function	GWO	GWO1	GWO2	GWO3	GWO4	FMGWO
7	2.38 × 10^−9^	7.56 × 10^−8^	1.14 × 10^−7^	9.16 × 10^−9^	1.70 × 10^−8^	3.69 × 10^−11^
8	3.28 × 10^−8^	5.07 × 10^−10^	6.25 × 10^−8^	1.74 × 10^−10^	1.93 × 10^−9^	2.15 × 10^−10^
9	−1.00	−1.00	−1.00	−1.00	−1.00	−1.00
10	0.00	0.00	0.00	0.00	0.00	0.00
11	−5.29 × 10^2^	−4.04 × 10^3^	−6.26 × 10^3^	−5.42 × 10^2^	−3.31 × 10^3^	−4.08 × 10^3^
12	−7.04 × 10^3^	−7.66 × 10^3^	−7.29 × 10^3^	−8.31 × 10^3^	−8.66 × 10^3^	−8.40 × 10^3^
13	−1.80	−1.80	−1.80	−1.80	−1.80	−1.80
14	−4.69	−4.65	−4.65	−4.69	−4.69	−4.69
15	−9.10	−9.58	−8.64	−9.26	−9.06	−9.16
16	0.00	0.00	0.00	0.00	0.00	0.00
17	−1.03	−1.03	−1.03	−1.03	−1.03	−1.03

**Table 10:** Average of each algorithm tested on F7–F17 functions.

Function	GWO	GWO1	GWO2	GWO3	GWO4	FMGWO
7	5.24 × 10^−7^	1.12 × 10^−7^	2.24 × 10^−7^	1.53 × 10^−7^	2.23 × 10^−7^	1.84 × 10^−8^
8	5.07 × 10^−7^	1.22 × 10^−7^	1.05 × 10^−7^	1.63 × 10^−7^	1.30 × 10^−7^	2.00 × 10^−8^
9	−1.00	−1.00	−1.00	−1.00	−1.00	−1.00
10	0.00	0.00	0.00	0.00	0.00	0.00
11	−4.35 × 10^2^	−2.73 × 10^3^	−5.82 × 10^3^	−4.51 × 10^2^	−2.44 × 10^3^	−3.17 × 10^3^
12	−5.87 × 10^3^	−6.15 × 10^3^	−6.70 × 10^3^	−6.61 × 10^3^	−6.21 × 10^3^	−7.73 × 10^3^
13	−1.80	−1.80	−1.80	−1.80	−1.77	−1.80
14	−4.23	−4.41	−4.60	−4.44	−4.37	−4.66
15	−7.37	−7.96	−8.26	−7.95	−7.65	−8.84
16	5.01 × 10^−3^	3.57 × 10^−3^	0.00	2.59 × 10^−3^	8.85 × 10^−3^	0.00
17	−1.03	−1.03	−1.03	−1.03	−1.03	−1.03

**Table 11:** Standard deviation of each algorithm tested on F7–F17 functions.

Function	GWO	GWO1	GWO2	GWO3	GWO4	FMGWO
7	5.19 × 10^−7^	2.63 × 10^−8^	7.74 × 10^−8^	1.22 × 10^−7^	1.94 × 10^−7^	1.23 × 10^−8^
8	4.15 × 10^−7^	9.95 × 10^−8^	3.48 × 10^−8^	1.74 × 10^−7^	1.26 × 10^−7^	1.31 × 10^−8^
9	9.09 × 10^−7^	2.03 × 10^−7^	4.95 × 10^−8^	1.33 × 10^−7^	2.72 × 10^−7^	1.28 × 10^−8^
10	0.00	0.00	0.00	0.00	0.00	0.00
11	3.88 × 10^1^	6.48 × 10^2^	2.42 × 10^2^	8.10 × 10^1^	4.23 × 10^2^	3.40 × 10^2^
12	7.92 × 10^2^	1.21 × 10^3^	5.39 × 10^2^	1.46 × 10^3^	1.19 × 10^3^	3.02 × 10^2^
13	2.54 × 10^−6^	1.61 × 10^−6^	4.93 × 10^−7^	1.90 × 10^−6^	1.44 × 10^−1^	8.22 × 10^−8^
14	3.71 × 10^−1^	3.04 × 10^−1^	5.72 × 10^−2^	3.05 × 10^−1^	3.58 × 10^−1^	2.01 × 10^−2^
15	1.05	1.27	2.40 × 10^−1^	8.45 × 10^−1^	8.36 × 10^−1^	1.65 × 10^−1^
16	4.77 × 10^−3^	4.68 × 10^−3^	0.00	4.30 × 10^−3^	2.64 × 10^−3^	0.00
17	3.13 × 10^−8^	4.04 × 10^−9^	1.98 × 10^−9^	3.24 × 10^−9^	7.35 × 10^−9^	6.74 × 10^−10^

**Table 12:** Worst values of each algorithm tested on F7–F17 functions.

Function	GWO	GWO1	GWO2	GWO3	GWO4	FMGWO
7	2.10 × 10^−6^	1.59 × 10^−7^	3.65 × 10^−7^	4.31 × 10^−7^	7.67 × 10^−7^	3.74 × 10^−8^
8	1.55 × 10^−6^	3.26 × 10^−7^	1.95 × 10^−7^	7.10 × 10^−7^	4.98 × 10^−7^	4.16 × 10^−8^
9	−1.00	−1.00	−1.00	−1.00	−1.00	−1.00
10	0.00	0.00	0.00	0.00	0.00	0.00
11	−3.30 × 10^2^	−1.24 × 10^3^	−5.41 × 10^3^	−2.15 × 10^2^	−1.69 × 10^3^	−2.77 × 10^3^
12	−4.37 × 10^3^	−4.02 × 10^3^	−5.49 × 10^3^	−3.50 × 10^3^	−3.57 × 10^3^	−7.30 × 10^3^
13	−1.80	−1.80	−1.80	−1.80	−1.00	−1.80
14	−3.50	−3.65	−4.50	−3.50	−3.54	−4.65
15	−4.48	−5.22	−7.90	−5.22	−5.67	−8.53
16	9.72 × 10^−3^	9.72 × 10^−3^	0.00	9.72 × 10^−3^	9.72 × 10^−3^	0.00
17	−1.03	−1.03	−1.03	−1.03	−1.03	−1.03

**Table 13:** Optimal values of each algorithm tested on F18–F33 functions.

Function	GWO	GWO1	GWO2	GWO3	GWO4	FMGWO
18	1.50 × 10^−9^	3.25 × 10^−9^	3.26 × 10^−8^	6.11 × 10^−10^	5.86 × 10^−10^	5.23 × 10^−12^
19	7.48 × 10^−5^	1.52 × 10^−4^	2.35 × 10^−3^	2.80 × 10^−6^	1.33 × 10^−3^	1.14 × 10^−5^
20	9.98 × 10^−1^	9.98 × 10^−1^	9.98 × 10^−1^	9.98 × 10^−1^	9.98 × 10^−1^	9.98 × 10^−1^
21	0.00	0.00	0.00	0.00	0.00	0.00
22	0.00	0.00	0.00	0.00	0.00	0.00
23	−1.87 × 10^2^	−1.87 × 10^2^	−1.87 × 10^2^	−1.87 × 10^2^	−1.87 × 10^2^	−1.87 × 10^2^
24	3.00	3.00	3.00	3.00	3.00	3.00
25	3.16 × 10^−4^	3.08 × 10^−4^	3.08 × 10^−4^	3.08 × 10^−4^	3.08 × 10^−4^	3.08 × 10^−4^
26	−1.04 × 10^1^	−1.04 × 10^1^	−1.04 × 10^1^	−1.04 × 10^1^	−1.04 × 10^1^	−1.04 × 10^1^
27	3.36 × 10^−3^	3.24 × 10^−4^	1.32 × 10^−2^	1.81 × 10^−3^	9.30 × 10^−4^	5.36 × 10^−4^
28	0.00	0.00	0.00	0.00	0.00	0.00
29	−3.86	−3.86	−3.86	−3.86	−3.86	−3.86
30	−3.32	−3.32	−3.32	−3.32	−3.32	−3.32
31	7.77 × 10^−5^	2.85 × 10^−7^	4.43 × 10^−4^	1.64 × 10^−5^	1.74 × 10^−5^	4.42 × 10^−6^
32	7.83 × 10^−2^	1.79 × 10^−2^	5.42 × 10^−1^	1.31 × 10^−2^	3.84 × 10^−2^	8.44 × 10^−3^
33	1.16 × 10^2^	9.51 × 10^1^	6.59 × 10^2^	6.77 × 10^1^	2.47 × 10^2^	2.06 × 10^1^

**Table 14:** Average of each algorithm tested on F18–F33 functions.

Function	GWO	GWO1	GWO2	GWO3	GWO4	FMGWO
18	2.31 × 10^−7^	3.74 × 10^−8^	5.67 × 10^−8^	4.72 × 10^−8^	5.08 × 10^−2^	5.19 × 10^−9^
19	1.15	1.65	2.40 × 10^−2^	7.66 × 10^−1^	3.39	2.34 × 10^−3^
20	5.82	1.04	9.98 × 10^−1^	1.21	6.28	9.98 × 10^−1^
21	0.00	0.00	0.00	0.00	0.00	0.00
22	0.00	0.00	0.00	0.00	0.00	0.00
23	−1.87 × 10^2^	−1.87 × 10^2^	−1.87 × 10^2^	−1.86 × 10^2^	−1.87 × 10^2^	−1.87 × 10^2^
24	3.00	3.00	3.00	3.00	8.43	3.00
25	9.10 × 10^−3^	1.13 × 10^−3^	3.08 × 10^−4^	1.16 × 10^−3^	2.24 × 10^−3^	3.08 × 10^−4^
26	−1.04 × 10^1^	−1.04 × 10^1^	−1.04 × 10^1^	−9.80	−9.62	−1.04 × 10^1^
27	9.17	2.52 × 10^−1^	3.68 × 10^−2^	2.06 × 10^−1^	1.27	7.73 × 10^−3^
28	0.00	0.00	0.00	0.00	0.00	0.00
29	−3.86	−3.86	−3.86	−3.86	−3.86	−3.86
30	−3.25	−3.25	−3.32	−3.28	−3.28	−3.32
31	2.57 × 10^−2^	8.93 × 10^−3^	7.38 × 10^−4^	2.14 × 10^−3^	2.75 × 10^−4^	5.90 × 10^−5^
32	8.57 × 10^1^	7.29 × 10^1^	3.61 × 10^1^	2.30 × 10^2^	1.26 × 10^2^	2.35 × 10^−1^
33	5.23 × 10^3^	2.98 × 10^3^	1.04 × 10^3^	3.27 × 10^3^	3.01 × 10^3^	2.89 × 10^2^

**Table 15:** Standard deviation of each algorithm tested on F18–F33 functions.

Function	GWO	GWO1	GWO2	GWO3	GWO4	FMGWO
18	1.96 × 10^−7^	3.87 × 10^−8^	1.62 × 10^−8^	3.82 × 10^−8^	1.90 × 10^−1^	4.44 × 10^−9^
19	1.31	2.13	3.06 × 10^−2^	8.13 × 10^−1^	2.92	2.94 × 10^−3^
20	4.42	1.78 × 10^−1^	1.26 × 10^−11^	6.45 × 10^−1^	4.27	5.64 × 10^−12^
21	0.00	0.00	0.00	0.00	0.00	0.00
22	0.00	0.00	0.00	0.00	0.00	0.00
23	2.35 × 10^−1^	1.43 × 10^−1^	4.58 × 10^−4^	1.02	1.67 × 10^−1^	2.44 × 10^−5^
24	4.35 × 10^−5^	1.32 × 10^−5^	4.50 × 10^−7^	9.44 × 10^−6^	2.02 × 10^1^	2.54 × 10^−7^
25	9.85 × 10^−3^	2.28 × 10^−3^	9.82 × 10^−8^	3.62 × 10^−3^	4.11 × 10^−3^	5.58 × 10^−9^
26	9.42 × 10^−4^	2.54 × 10^−4^	1.57 × 10^−4^	1.85	2.03	7.08 × 10^−5^
27	4.06 × 10^1^	3.18 × 10^−1^	1.78 × 10^−2^	2.23 × 10^−1^	1.38	6.97 × 10^−3^
28	0.00	0.00	0.00	0.00	0.00	0.00
29	1.49 × 10^−3^	2.07 × 10^−4^	3.43 × 10^−6^	3.05 × 10^−4^	3.04 × 10^−3^	3.40 × 10^−7^
30	8.00 × 10^−2^	5.92 × 10^−2^	5.43 × 10^−6^	5.78 × 10^−2^	9.75 × 10^−2^	4.51 × 10^−7^
31	5.57 × 10^−2^	2.84 × 10^−2^	1.85 × 10^−4^	8.85 × 10^−3^	2.19 × 10^−4^	4.07 × 10^−5^
32	8.44 × 10^1^	9.52 × 10^1^	5.76 × 10^1^	4.88 × 10^2^	2.04 × 10^2^	2.42 × 10^−1^
33	8.44 × 10^3^	4.15 × 10^3^	3.33 × 10^2^	4.23 × 10^3^	3.59 × 10^3^	1.85 × 10^2^

**Table 16:** Worst values of each algorithm tested on F18–F33 functions.

Function	GWO	GWO1	GWO2	GWO3	GWO4	FMGWO
18	8.45 × 10^−7^	1.48 × 10^−7^	8.94 × 10^−8^	1.48 × 10^−7^	7.62 × 10^−1^	1.36 × 10^−8^
19	6.86	7.84	1.08 × 10^−1^	2.09	7.88	1.08 × 10^−2^
20	1.27 × 10^1^	1.99	9.98 × 10^−1^	3.97	1.55 × 10^1^	9.98 × 10^−1^
21	0.00	0.00	0.00	0.00	0.00	0.00
22	0.00	0.00	0.00	0.00	0.00	0.00
23	−1.86 × 10^2^	−1.86 × 10^2^	−1.87 × 10^2^	−1.81 × 10^2^	−1.86 × 10^2^	−1.87 × 10^2^
24	3.00	3.00	3.00	3.00	8.40 × 10^1^	3.00
25	2.04 × 10^−2^	8.97 × 10^−3^	3.08 × 10^−4^	2.04 × 10^−2^	1.29 × 10^−2^	3.08 × 10^−4^
26	−1.04 × 10^1^	−1.04 × 10^1^	−1.04 × 10^1^	−2.77	−2.77	−1.04 × 10^1^
27	2.28 × 10^2^	1.27	6.66 × 10^−2^	7.71 × 10^−1^	5.45	2.40 × 10^−2^
28	0.00	0.00	0.00	0.00	0.00	0.00
29	−3.85	−3.86	−3.86	−3.86	−3.85	−3.86
30	−3.08	−3.20	−3.32	−3.20	−2.84	−3.32
31	2.11 × 10^−1^	1.46 × 10^−1^	1.06 × 10^−3^	4.91 × 10^−2^	8.11 × 10^−4^	1.38 × 10^−4^
32	2.14 × 10^2^	3.42 × 10^2^	1.62 × 10^2^	2.37 × 10^3^	1.09 × 10^3^	7.57 × 10^−1^
33	4.08 × 10^4^	2.07 × 10^4^	1.59 × 10^3^	1.90 × 10^4^	1.83 × 10^4^	5.70 × 10^2^

**Table 17:** Parameter setting of wireless sensor network coverage experiment.

Parameter	Value
Monitoring area	100 m × 100 m
Number of nodes	20/25/30
Node perception radius r	12
Total number of iterations T	500
Number of grids	100 × 100

**Table 18:** Initialization parameters of all algorithms.

Algorithms	Parameter Settings
PSO	N = 30, ω = 0.8, c1 = 2, c2 = 2
GWO	N = 30
CSA	AP = 0.1, FL = 2
DE	F = 0.5, CR = 0.9
GA	P_c_ = 0.8, P_m_ = 0.1
FA	α = 0.2, β_0_ = 1, γ = 0.00001
OGWO	N = 30, μ = 2, J_r_ = 0.05
DGWO1	N = 30
DGWO2	N = 30
FMGWO	N = 30

**Table 19:** Comparison data of WSN application experiments.

Algorithms	Performance Index	Nodes 20	Nodes 25	Nodes 30
PSO	Best	78.98%	85.85%	91.55%
	Mean	75.32%	83.24%	88.92%
	Std	0.014766	0.014151	0.013767
GWO	Best	83.17%	94.56%	98.07%
	Mean	81.37%	91.48%	95.90%
	Std	0.011705	0.014196	0.030498
CSA	Best	72.61%	81.86%	86.25%
	Mean	70.38%	78.98%	84.46%
	Std	0.009383	0.01174	0.008077
DE	Best	84.03%	87.94%	91.36%
	Mean	70.78%	77.64%	84.39%
	Std	0.040835	0.025091	0.028479
GA	Best	75.11%	83.64%	89.98%
	Mean	72.37%	81.00%	87.03%
	Std	0.011797	0.012978	0.010928
FA	Best	68.92%	79.48%	85.26%
	Mean	66.43%	75.46%	82.55%
	Std	0.013747	0.01946	0.012655
OGWO	Best	82.53%	92.67%	98.29%
	Mean	70.66%	80.27%	85.58%
	Std	0.060519	0.069433	0.065456
DGWO1	Best	83.98%	94.03%	98.15%
	Mean	81.89%	91.69%	96.09%
	Std	0.013674	0.010031	0.027406
DGWO2	Best	82.67%	92.95%	96.98%
	Mean	78.47%	88.56%	94.46%
	Std	0.020177	0.023526	0.014734
FMGWO	Best	84.39%	94.85%	98.63%
	Mean	82.47%	92.24%	96.57%
	Std	0.0092	0.025269	0.031685

## Data Availability

Data are contained within the article.
